# Open-source automated centrifugal pump test rig

**DOI:** 10.1016/j.ohx.2020.e00140

**Published:** 2020-09-11

**Authors:** Clayton S. Semenzin, Martin Mapley, Eric Wu, Jo P. Pauls, Benjamin Simpson, Shaun D. Gregory, Geoff Tansley

**Affiliations:** a School of Engineering and Built Environment, Griffith University, Southport, Australia; b The Innovative Cardiovascular Engineering and Technology Laboratory, Critical Care Research Group, The Prince Charles Hospital, Chermside, Australia; c School of Medicine, University of Queensland, Brisbane, Australia; d Department of Engineering, Nottingham Trent University, Nottingham, UK; e Department of Mechanical and Aerospace Engineering, Monash University, Melbourne, Australia

**Keywords:** Performance curves, Efficiency curves, Small pumps

## Abstract

Design methods for large industrial pumps are well developed, but they cannot be relied upon when designing specialised miniature pumps, due to scaling issues. Therefore, the design and development phase of small pumps demand numerous experimental tests to ensure a viable prototype. Of initial interest is hydraulic design in the form of pump performance and efficiency curves. This project aimed to produce an automated test rig capable of generating both the performance (P-Q – pressure vs. flow rate) and efficiency curves that are reliable and repeatable. The apparatus is largely customizable and suitable for a range of smaller pump sizes. The pump impeller and volute were 3D printed, allowing for design flexibility and rapid prototyping and testing.

The test loop was automated which allowed the flow rate to be incremented from 0 L/min to the maximum flow rate. At each step the pressure, flow rate, voltage and current were recorded to generate the P – Q and efficiency curves. Repeatability results showed low variations of ±3 mmHg (400 Pa) in pressure and ± 2% in hydraulic efficiency. The given setup can be used to compare and evaluate the hydraulic performance of various pump designs.

## Graphical abstract

**Figure:**
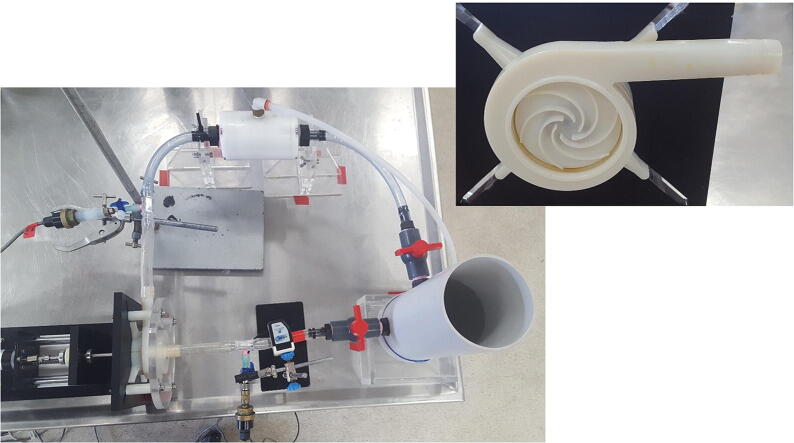

## 1 Introduction

### 1.1 Specifications table

**Table:** 

Hardware name	*Open-Source Automated Centrifugal Pump Test Rig*
Subject area	• Fluid Dynamics • Mechanical Engineering • General
Hardware type	• Fluidic Handling • Electrical engineering • Mechanical engineering
Open Source License	CC by 4.0
Cost of Hardware	*$* 4 160
Source File Repository	https://doi.org/10.17605/OSF.IO/9T2Z6

## 2 Hardware in context

When developing pumps, it is common to follow traditional pump design theory such as the methodology proposed by Stepanoff in 1948 [1]. Traditional pump theories rely on design constants derived for industrial pumping applications, which presents scaling down issues when applied to much smaller pumps, leading to a less than optimal design [2], [3], [4]. The performance characteristics are typically improved upon through a building-and-testing approach as there is no pure theoretical or analytical solution to determine the hydraulic performance of pumps [1]. Therefore, the optimisation of small pumps must be performed experimentally and iteratively.

A number of design variables influence the performance of pumps. Generally considered the single most important component is the impeller. Design variations of the impeller can include altering the impeller diameter, blade angles, blade heights and the number of blades. The coupling between the impeller and volute are also highly influential. The volute design is largely dictated by the ‘spiral’ shape, affecting the pressure and velocity of the collecting flow leaving the impeller. Another interaction that has significant impact on performance is the axial clearance. This represents the gap between the top of the impeller blades and the pump housing, influencing the flow leakage over the impeller blades; the pressure generated and pump efficiency are highly sensitive to this clearance [5]. The presented test rig accommodates for the mentioned variables, resulting in a large number of design variations.

The two measures of primary importance for characterizing the performance of these pumps are the P – Q (pressure vs. flow rate) curves and efficiency curves. The pressure used in the P – Q curves is defined as the static pressure difference between the outlet and inlet pump terminals. The ideal design or operating point for a pump should coincide with the best efficiency point. The presented hardware was used to evaluate blood pump designs where the design point is typically concentrated around a pressure value of 100 mmHg and a flow rate of 5 L/min. In this application it is important to maximise the efficiency at this operating point [5].

## 3 Hardware description

The presented automated pump test rig was developed to assess the performance of various blood pump designs. The following sections give a brief description of the major components.

### 3.1 Test loop

**Fig. 1:**
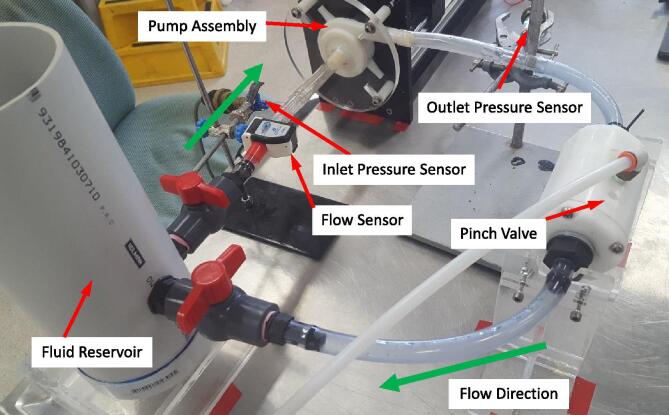
Test loop main components.

A simple test loop allows fluid to circulate through the pump while monitoring the pressure difference and flow rate. In order to generate the performance and efficiency curves the pressure, voltage and current are recorded at various flow rates (keeping constant pump speeds). The flow rate is manipulated using a PWM driven air regulator (ITV2030-312BS5, SMC, Tokyo, Japan) (not shown) to adjust a pneumatic pinch valve (Fig. 1). For each experiment the flow rate starts at 0 L/min and increases by 0.5 L/min every 75 s. This can be altered through the provided SimuLink file. The changing target flow signal was generated using Simulink (R2015a, Mathworks, Natick, MA, USA) code. It included a proportional-integral-derivative (PID) controller, ensuring the flow rate reached and settled at the target flow, using the flow sensor as feedback, before incrementing to the next step. The working fluid consisted of a water – glycerol solution to approximate the fluid properties of blood.

### 3.2 Test rig

**Fig. 2:**
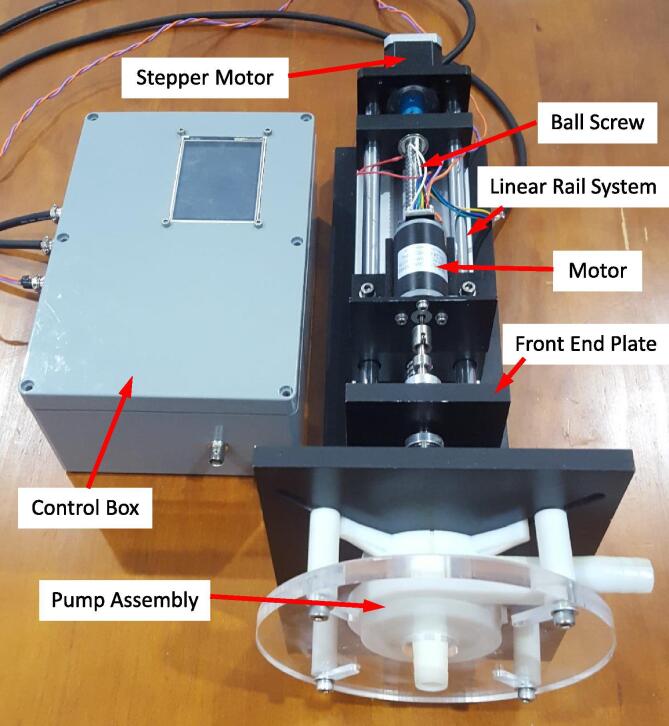
Test rig main components.

**Fig. 3:**
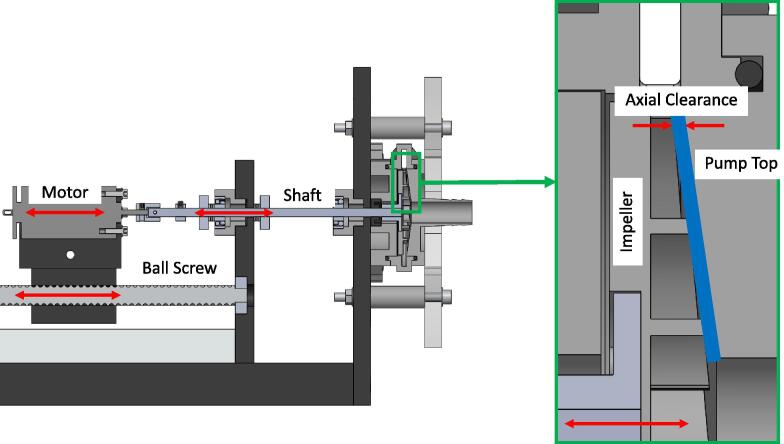
Cut-away showing adjustment of the axial clearance.

The test rig consists of a DC motor mounted to a linear rail system. The original front end plate was replaced with a custom plate that included a bearing to support the shaft (Fig. 2). Using a stepper motor to drive the ball screw, the position of the motor and shaft can be controlled, changing the axial clearance within the pump (Fig. 3).

### 3.3 Pump assembly

**Fig. 4:**
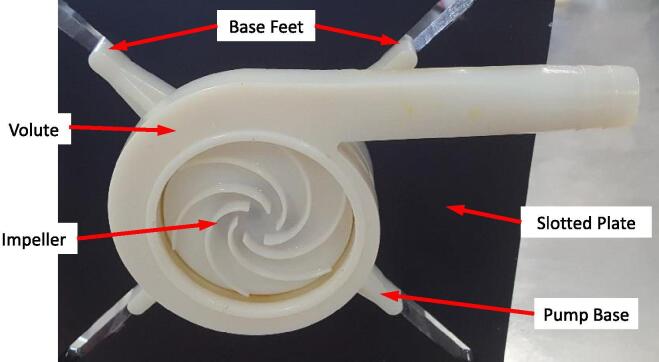
Pump assembly components.

The pump assembly comprises four components: the pump base, volute, impeller and pump top (not shown). These components are all 3D printed which allows for rapid prototyping of various designs. The pump design is largely customisable with the only requirement being that the base feet are located in the slotted plate (Fig. 4) to centre the shaft in the volute.

All 3D printing was performed in-house (Objet30 Prime, Stratasys, Rehovot, Israel) using VeroWhitePlus (RGD835, Stratasys, Rehovot, Israel) as the model material and SUP706 B (SUP706 B, Stratasys, Rehovot, Israel) as the support. It is understood that the 3D printed parts will bear a higher surface roughness than commercial pumps. Rougher surfaces will influence the pump losses and hence, impact the measured performance. The purpose of the automated test rig is to quickly identify the most promising prototype – each of which will have performance affected to the same degree and relative performance will be unaffected. To reduce the impact of surface roughness, all components were produced using the finest print settings and oriented to ensure the smoothest possible finish on key surfaces. The surface roughness (Sa) was measured to be 0.05 μm using a Zeta300 optical profilometer (KLA-Tencor, Milpitas, CA, USA). The influence of surface roughness is expected to me minimal compared to the contribution of other experimental factors.

### 3.4 Control box

**Fig. 5:**
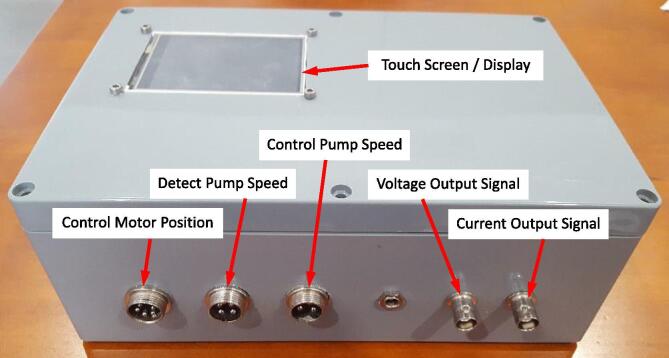
Control box components.

A touch screen is utilized to allow on-the-fly manipulation of the pump speed and positioning of the motor assembly on the linear rail system (Fig. 5). The control box makes use of an Arduino Due (Arduino Due R3, Arduino SRL, Scarmagno, Italy) incorporating programming for a PID controller to accurately set and maintain the motor speed. The display outputs a readout showing the target and measured speed as well as the relative current position of the linear rail platform. The control box is responsible for sampling the voltage and current (signal output to the data acquisition system via an LM324N op amp) used by the motor for efficiency calculations. Component details are available in the Bill of Materials.

### 3.5 Data acquisition – Control Desk / dSPACE

In this system a dSPACE (DS1104, dSPACE, Paderborn, Germany) data acquisition module was used to read all data signals (pressure, flow rate, voltage and current) and send the appropriate signal to the air regulator for pinch valve (flow rate) adjustment. The dSPACE system was used as it was readily available, however, any DAQ could perform these tasks. Control Desk is the program that gives the dSPACE a user interface. Some adjustment to the calibration and tuning built into the SimuLink code can be made from Control Desk. This includes the flow rate increments, timing and PID gains. The program provides real-time visualisation of the data and access to the controls necessary to setup/run the experiment.

## 4 Design files

### 4.1 Design files summary

**Table:** 

Design file name	File type	Open source license	Location of the file
Base_Plate	CAD	CC by 4.0	https://doi.org/10.17605/OSF.IO/9T2Z6
Bearing_Plate	CAD	CC by 4.0	https://doi.org/10.17605/OSF.IO/9T2Z6
Motor_Mount	CAD	CC by 4.0	https://doi.org/10.17605/OSF.IO/9T2Z6
Slotted_Plate	CAD	CC by 4.0	https://doi.org/10.17605/OSF.IO/9T2Z6
Shaft	CAD	CC by 4.0	https://doi.org/10.17605/OSF.IO/9T2Z6
Base_Stand	CAD	CC by 4.0	https://doi.org/10.17605/OSF.IO/9T2Z6
Bracket_Stand	CAD	CC by 4.0	https://doi.org/10.17605/OSF.IO/9T2Z6
Centre_Section	CAD	CC by 4.0	https://doi.org/10.17605/OSF.IO/9T2Z6
Slide_Section	CAD	CC by 4.0	https://doi.org/10.17605/OSF.IO/9T2Z6
Bearing_Adapter	CAD	CC by 4.0	https://doi.org/10.17605/OSF.IO/9T2Z6
Pump_Base	CAD	CC by 4.0	https://doi.org/10.17605/OSF.IO/9T2Z6
Pump_Top	CAD	CC by 4.0	https://doi.org/10.17605/OSF.IO/9T2Z6
Impeller	CAD	CC by 4.0	https://doi.org/10.17605/OSF.IO/9T2Z6
Volute	CAD	CC by 4.0	https://doi.org/10.17605/OSF.IO/9T2Z6
Spacers	CAD	CC by 4.0	https://doi.org/10.17605/OSF.IO/9T2Z6
Wiring	Image	CC by 4.0	https://doi.org/10.17605/OSF.IO/9T2Z6
SimuLink	SimuLink	CC by 4.0	https://doi.org/10.17605/OSF.IO/9T2Z6
Arduino	Arduino	CC by 4.0	https://doi.org/10.17605/OSF.IO/9T2Z6
MatLab	MatLab script	CC by 4.0	https://doi.org/10.17605/OSF.IO/9T2Z6

* All CAD files are provided as SolidWorks files (.sldprt) to allow for customisation as well as a neutral format for the machined parts (.step), laser cut sections (.dxf) and 3D printed components (.stl).

• Pump Test Rig – Contains the various machined components required to construct the test rig. Note all threads are cosmetic and standard sizes were used.
• Stand – Folder containing the CAD files used for the pinch valve stands. These components were all laser cut.
• Pump Assembly – This folder consists of the 3D printed models used to prototype the pump. These files can be modified to assess the performance of alternative pump designs.
• Wiring – High resolution image of the electrical wiring diagram used to construct the control box.
• SimuLink - .slx file developed to automate the flow rate increase during the experiment. Contains editable blocks for tuning and calibration.
• Arduino – sketch uploaded to the Arduino Due for pump speed control and linear rail platform positioning.
• MATLAB – MATLAB script used to extract data and generate pump performance and efficiency curves.

## 5 Bill of materials

### 5.1 Bill of materials

**Table:** 

**Designator**	**Component**	**Number**	**Cost per unit -currency**	**Total cost -currency**	**Source of materials**	**Material type**
**Electrical**						
Computer	PC	1			Recycled	Other
Power Supply	Minleaf DC 60 V 5A Adjustable power supply	1	$99.99	$99.99	ebay.com.au	Other
dSPACE	DS1104	1			Recycled	Other
Electrical Box	240 × 160 × 90 mm Electrical Box (HB6134)	1	$39.95	$39.95	Jaycar	ABS
Arduino	Arduino Due (with headers) R3 MCU development board A000062 (769–7412)	1	$57.52	$57.52	RS online	Other
Touch Screen	3.2″ TFT Touch screen LCD display + Mega Shield V2.2	1	$26.80	$26.80	ebay.com.au	Other
Stepper Motor Driver	TB6560 Stepper motor driver	1	$19.95	$19.95	ebay.com.au	Other
12 V Voltage Regulator	MC78T12 12 V 3A Voltage Regulator (ZV1634)	1	$4.95	$4.95	Jaycar	Composite
Regulator Heatsink	Regulator Heatsink	1			Recycled	Metal
MOSFET	Infineon IRL520NPBF MOSFET (541–1196)	1	$1.81	$1.81	RS online	Composite
MOSFET Heatsink	MOSFET Heatsink	1			Recycled	Metal
Op Amps	LM324 Quadruple Op Amp (ZL3324)	1	$2.35	$2.35	Jaycar	Composite
Diode	1 N4004 Diode (ZR1004)	1	$0.98 (pack of 4)	$0.98	Jaycar	Metal
15 kΩ Resistor	15kΩ ± 1% 0.5 W metal film resistor (RR0600)	3	$0.55 (pack of 8)	$0.55	Jaycar	Composite
27 kΩ Resistor	27kΩ ± 1% 0.5 W metal film resistor (RR0606)	4	$0.55 (pack of 8)	$0.55	Jaycar	Composite
150 Ω Resistor	150 Ω ± 1% 0.5 W metal film resistor (RR0552)	1	$0.85 (pack of 8)	$0.85	Jaycar	Composite
82 kΩ Resistor	82kΩ ± 1% 0.5 W metal film resistor (RR0618)	4	$0.55 (pack of 8)	$0.55	Jaycar	Composite
3.9 kΩ Resistor	3.9kΩ ± 1% 0.5 W metal film resistor (RR0586)	2	$0.55 (pack of 8)	$0.55	Jaycar	Composite
Current Sense Resistor	Vishay wirewound 0.1 Ω ± 1% 3 W resistor LVR03 series (LVR03R1000FE70)	1	$3.51	$3.51	Element14	Composite
Motor	Chihai 24 V 8000 rpm motor with quadrature hall effect sensor (CHR-RS3162)	1	$15.45	$15.45	DX.com	Metal
5 Pin Socket	GX-16 5 Pin Female (PS2018)	1	$3.75	$3.75	Jaycar	Metal
5 Pin Plug	GX-16 5 Pin Male (PP2017)	1	$3.50	$3.50	Jaycar	Metal
4 Pin Socket	GX-16 4 Pin Female (PS2012)	1	$3.75	$3.75	Jaycar	Metal
4 Pin Plug	GX-16 4 Pin Male (PP2010)	1	$3.50	$3.50	Jaycar	Metal
2 Pin Socket	GX-16 2 Pin Female (PS2014)	1	$3.75	$3.75	Jaycar	Metal
2 Pin Plug	GX-16 2 Pin Male (PP2013)	1	$3.50	$3.50	Jaycar	Metal
DC Power Plug	2.5 mm DC Power Line Connector 10 mm Shaft (PP0511)	1	$2.25	$2.25	Jaycar	Metal
DC Power Socket	2.5 mm Bulkhead Male DC Power Connector (PS0524)	1	$2.95	$2.95	Jaycar	Metal
BNC connector	RS Pro 75 Ω Panel Mount Bulkhead BNC Connector, Solder Termination (546–4904)	2	$3.98	$7.96	RS online	Metal
Banana Plug – Black	Black Right Angle Banana Plug (PP0395)	1	$2.50	$2.50	Jaycar	Composite
Banana Plug – Red	Red Right Angle Banana Plug (PP0394)	1	$2.50	$2.50	Jaycar	Composite
2 Core Power Cable	7.5A 2 core Tinned DC Power cable (WH3057)	2 m	$1.60/m	$3.20	Jaycar	Composite
4 Core Cable	4 Core Screened Microphone Cable (WB1540)	2 m	$3.45/m	$6.90	Jaycar	Composite
BNC Cable	2.0 Lead, BNC PLG-PLG, 50R(779829–58-2.0)	2	$6.95	$13.90	Element 14	Composite
Electrical Wiring	Hook-Up Wire Pack – 2 m, 8 colours (WH3025)	5 m	$5.95	$5.95	Jaycar	Composite
Prototype Board	Small Breadboard Layout Prototyping Board (HP9570)	1	$4.95	$4.95	Jaycar	Other
**Mechanical**						
Linear Rail System	CNC GGP Ball Screw 1204 200 mm Stroke Linear Motion Guide Rail + 23 NEMA Stepper Motor	1	$120.22	$120.22	aliexpress.com	Metal
8 mm Bearing	8 mm Deep Groove Ball Bearing 22 mm OD 608-2Z(137–1471)	1	$7.23	$7.23	RS online	Metal
Coupling Adapter	Coupling adapter	1	In-house		Objective 3D	VeroWhitePlusSup706 B
Cup Joint	HPI Heavy Duty Cup Joint 5 × 10 × 16 mm	1	$14.99	$14.99	ebay.com.au	Metal
Roll Pin	300 Grade Stainless Steel 2 mm × 16 mm Length Roll Pin(RP020-0160–4)	1	$2.68	$2.68	Small Parts and Bearings	Metal
Small Shaft Collar	RS Pro One Piece Screw Collar, 5 mm Bore, Stainless Steel (122–3448)	1	$5.23 (pack of 2)	$5.23	RS online	Stainless steel
Large Shaft Collar	Ruland Shaft Collar One Piece Clamp Screw, 5 mm Bore, Aluminium ENCL20-5MM-A (174–6673)	2	$13.29	$26.58	RS online	Aluminium
Bearing Adapter	Bearing Adapters	2	In-house		Objective 3D	VeroWhitePlusSup706 B
Self-Aligning Bearings	VXB 135 Self-Aligning Bearing 5 × 19 × 6 Miniature (12124)	2	$15.28	$30.56	ebay.com.au	Chrome Steel
Thrust Bearings	Align F5-10 M Axial Thrust Bearings (HN6125)	2	$12.20	$24.40	ebay.com.au	Stainless steel
**Machined Parts**						
Base Plate	Base Plate	1	$600	Total cost for all parts	Griffith University	Aluminium
Bearing Plate	Bearing Plate	1			Griffith University	Aluminium
Slotted Plate	Slotted Plate	1			Griffith University	Aluminium
Motor Mount	Motor Mount	1			Griffith University	Aluminium
Shaft	Shaft	1			Griffith University	Stainless Steel
Anodising	Anodising	1	$50.00	$50.00	A Grade Anodising	Aluminium
**Fasteners**						
M6 × 25 mm Bolts	M6 × 25 mm Socket Head Cap Screws Zinc-Plated (SHCSM625ZP)	6	$0.51	$3.06	Bolts and Industrial Supplies	Metal
M6 × 20 mm Bolts	M6 × 20 mm Socket Head Cap Screws Zinc-Plated (SHCSM620ZP)	4	$0.51	$2.04	Bolts and Industrial Supplies	Metal
M5 × 10 mm Bolts	M5 × 10 mm Socket Head Cap Screws Zinc-Plated (SHCSM510Z)	4	$0.37	$1.48	Bolts and Industrial Supplies	Metal
M3 × 10 mm Bolts	M3 × 10 mm Socket Head Cap Screws Zinc-Plated (SHCSM310Z)	4	$0.58	$2.32	Bolts and Industrial Supplies	Metal
Button M3 × 8 mm Bolts	M3 × 8 mm Button Head Cap Screws (BHCSM38)	20	$0.67	$13.40	Bolts and Industrial Supplies	Metal
M3 × 16 mm Bolts	M3 × 16 mm Socket Head Cap Screws Zinc-Plated (SHCSM316Z)	4	$0.54	$2.16	Bolts and Industrial Supplies	Metal
Button M3 × 6 mm Bolt	Button M3 × 6 mm Button Head Cap Screws Zinc Plated (BHCSM36Z)	1	$1.22	$1.22	Bolts and Industrial Supplies	Metal
M5 × 65 mm Bolts	Hobson Socket Head Cap Screw M5 × 65 mm Stainless Steel G304	4	$9.00 (pack of 5)	$9.00	ebay.com.au	Metal
M3 × 3 mm Grub Screw	M3 × 3 mm Steel Hex Head Cup Point Grub Screw	1	$3.69 (pack of 5)	$3.69	ebay.com.au	Steel
M3 × 2 mm Grub Screw	M3 × 2 mm Steel Hex Head Cup Point Grub Screw	1	$3.69 (pack of 5)	$3.69	ebay.com.au	Steel
M3 Nuts	M3 G316 Stainless Steel Std Hex Nuts (SSHN316M3)	4	$0.20	$0.80	Bolts and Industrial Supplies	Stainless Steel
M5 Nuts	M5 G316 Stainless Steel Std Hex Nuts (SSHN316M5)	4	$0.11	$0.44	Bolts and Industrial Supplies	Stainless Steel
M5 Washers	Zenith M5 316 Stainless Steel Flat Washer – 25 Pack(2330001)	16	$4.70 (pack of 25)	$4.70	Bunnings	Stainless Steel
M3 Washers	Zenith M3 316 Stainless Steel Flat Washer – 35 Pack(2320123)	4	$4.60 (pack of 35)	$4.60	Bunnings	Stainless Steel
4 mm Nylon Washers	4 mm Nylon Washer – Pk.25 (HP0166)	4	$5.00 (Pack of 25)	$5.00	Jaycar	Nylon
M3 × 25 mm Standoffs	Wurth Elektronik Brass Hex Standoff 970250324, 25 mm, M3 (184–2757)	10	$1.78	$17.80	RS online	Brass
M5 × 25 mm Bolts	M5 × 25 mm Socket Head Cap Screws Zinc-Plated (SHCSM525ZP)	4	$0.49	$1.96	Bolts and Industrial Supplies	Stainless Steel
M5 Wing Nuts	M5 G316 Stainless Steel Wing Nuts (SSWN316M5)	4	$0.95	$3.80	Bolts and Industrial Supplies	Stainless Steel
**Pump**						
Pump Base	Pump Base	1	In-house		Objective 3D	VeroWhitePlusSup706 B
Volute	Volute	1				
Pump Top	Pump Top	1				
Impeller	Impeller	1				
Spacers	Spacers	4				
52 × 2 mm O-rings	52 × 2 mm O-Ring NBR 70 Duro(N70M-0520020)	2	$2.16	$4.32	Ludowici Sealing Solutions	Nitrile Rubber
5 mm Rigid Flange Coupling	5 mm Rigid Flange Coupling Motor Guide Shaft Coupler	1	$2.94	$2.94	ebay.com.au	Metal
Acrylic Plate	Acrylic Plate	1	$33.00	$33.00	Laser Cut Designs	Acrylic
5 mm Shaft Seal	RS Pro Nitrile Rubber Seal, 5 mm Bore, 16 mm O.D (211–8715)	1	$17.08 (pack of 5)	$17.08	RS online	Nitrile Rubber
**Loop**						
½” Vinyl Tubing	Pope 13 mm × 5 m Clear Vinyl Tubing (3130568)	1	$20.91/5 m length	$20.91	Bunnings	Plastic
½” Tygon Tubing	Tygon E3603 - ACF00036 ID: 1/2″ OD: 5/8″ Wall: 1/16″ Length: 15 m	1	$235.10/ 15 m length	$235.10	Gallay Medical and Scientific	Plastic
Luer connector	½”× ½” Single Luer Connector (27226)	2	$3.57	$7.14	Qosina	Plastic
3-Way Taps	Stop Cock 3 Way Intravenous Tap (IV110001)	2	$2.10	$4.20	Solmed	Plastic
Pressure Transducers	Omega PX181B – 015C5VX346 Pressure Transducer	2	$369.00	$738.00	Omega Engineering	Other
PVC Pipe	Holman 100 mm × 1 m PVC DWV Pipe (4770090)	1	$15.00/(1 m length)	$15.00	Bunnings	Plastic
PVC Cap	Holman 100 mm PVC DWV Push on Cap (0149127)	1	$1.08	$1.08	Bunnings	Plastic
PVC Glue	Protek 250 mL Type N Blue PVC Cement (4750115)	1	$5.00	$5.00	Bunnings	Other
PVC Priming Fluid	Protek 125 mL Red Priming Fluid (4750121)	1	$4.50	$4.50	Bunnings	Other
20 mm Ball Valve	Holman 20 mm PVC Solvent Weld Ball Valve (5070624)	2	$9.85	$19.70	Bunnings	Plastic
¾ ” Brass Flange Nut	¾ ” BSP Wide Flange Brass Nut	2	$6.44 (5pk)	$6.44	ebay.com.au	Metal
¾” Tank Gasket	Philmac ¾” Tank Outlet (4814051)	2	$12.55 (for 1 outlet and 2 gaskets)	$12.55	Bunnings	Rubber
¾” Nipple	Garden Rain 0.75″ Poly Irrigation Nipple (3100150)	2	$2.08	$4.16	Bunnings	Plastic
18 mm O-ring	Holman 18 mm Rubber O-ring, 5 pack (3110573)	2	$4.45 (5pk)	$4.45	Bunnings	Rubber
25–13 mm Threaded to Barbed End	Pope 13 mm Tail × 25 mm BSP Male Director (3128229)	2	$1.15	$2.30	Bunnings	Plastic
20–13 mm Threaded to Barbed End	Pope 13 mm Tail × 20 mm BSP Male Director (3128211)	2	$1.15	$2.30	Bunnings	Plastic
Thread Tape	Kinetic 12 mm × 10 m Premium Thread Seal (4920187)	1	$3.40	$3.40	Bunnings	Other
Glycerol	Glycerol UNIVAR 2.5L GL(AJA242-2.5LGL)	1	$64.75/2.5 L	$64.75	Bacto Laboratories Pty Ltd	Other
Pinch Valve Stand	Pinch Valve Stand	2	$29.60	$59.20	Global Acrylic	Acrylic
Reservoir Stand	Reservoir Stand	1	$34.00	$34.00	Laser Cut Designs	Acrylic
Lab Stand	Mini Lab Bracket Retort Stand Clip Clamp 30 cm High	2	$19.49	$39.98	ebay.com.au	Metal
**Loop control**						
Pinch Valve	AKO VMP025.04 K.71Pinch Valve	1	$352.00	$352.00	CONVAIR Engineering Pty Ltd	Other
Manual air Regulator	SMC Precision Modular Regulator (IR3000-04)	1	$208.89*	$208.89*	SMC	Other
Voltage air Regulator	SMC Electro-Pneumatic Regulator(ITV2030-312BS5)	1	$653.28*	$653.28*	SMC	Other
3/8″ Soft Nylon Air Tube	SMC TIS Soft Nylon Tubing 3/8 OD, wht, 20 m (TISA11W-20)	1	$73.76* (20 m roll)	$73.76*	SMC	Plastic
PVC Air Hose	Kinetic 6 mm × 2 m Reinforced Pressure Hose (4920246)	1	$11.85/ (2 m length)	$11.85	Bunnings	Plastic
Push in Air Tube Connectors	SMC One Touch UNIFIT Male Connector(KQ2 L 11 – U04A)	2	$100.06* (pack of 10)	$100.06*	SMC	Composite
Air Hose Quick Connect	Craftright 6 Piece Nitto Style Fittings Set (6330410)	1	$26.95 (6 piece set)	$26.95	Bunnings	Metal
600PF Quick Connect	600PF Nitto Style Quick connect	1	$55.08	$55.08	ebay.com.au	Metal
¾” Male – ¾” Male connector	Brass Hex Nipple Male Threaded BSP, 20 mm (¾”)	1	$9.35	$9.35	Valve Warehouse Australia	Metal
¾” Male – ½” Male Connector	Brass Reducing Hex Nipple – Male × Male BSP, 25 × 15 mm (¾” × ½”)	1	$9.15	$9.15	Valve Warehouse Australia	Metal
½” Female – ½” Male Connector	Brass BSP Threaded Adaptor – Male & Female, 15 mm (½”)	1	$4.68	$4.68	Valve Warehouse Australia	Metal
Hose Clamps	Kinetic 11–25 mm 304 Stainless Steel Hose Clamp (0110737)	2	$1.50	$3.00	Bunnings	Metal
**Total = ~ $ 4160**						

All bolts are socket head unless otherwise specified.

* Prices were converted to AUD from USD then 10% added for GST.

Note: All prices are AUD and include GST (10%).

## 6 Build instructions

This section is focused on the assembly of the test setup. The tools required for assembly are commonly found in workshops and laboratories with the exception of a milling machine and laser cutter. The instructions outline those used to build the given setup with some simplified alternatives also suggested.

### 6.1 Test rig

The test rig is defined as the components used to drive the pump situated between the control box and pump assembly. Firstly, the aluminium sections (base plate, bearing plate, slotted plate, motor mount and shaft) should be machined according to the supplied CAD files.

1) Attach the bearing plate.

**Fig. 6:**
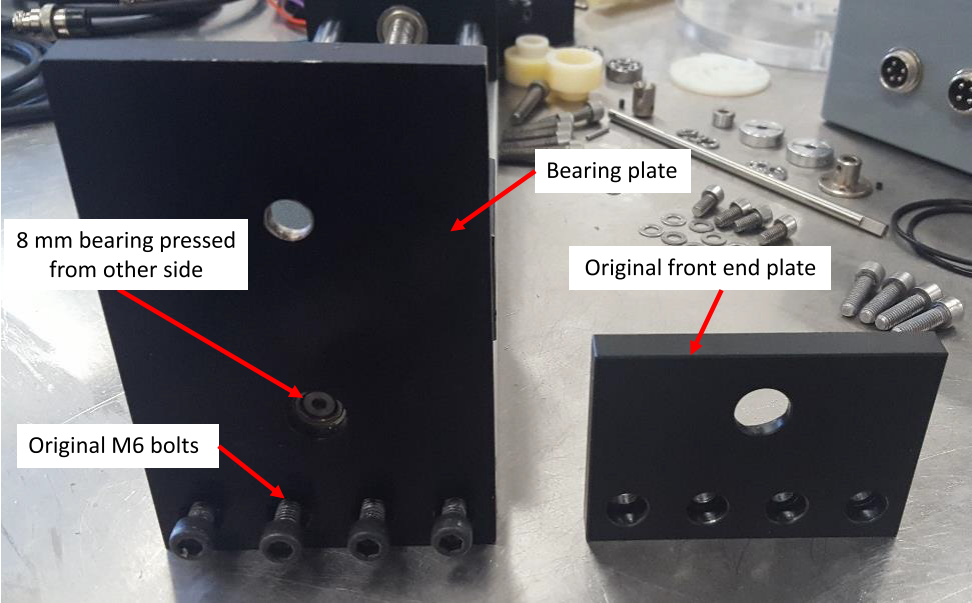
Replacement of the original front plate with the bearing plate.

Carefully remove the original front end plate of the linear rail system (Fig. 6) by removing the original M6 bolts. Press the 8 mm radial ball bearing into the recess that aligns with the ball screw on the bearing plate. Fix the bearing plate in place using the original M6 bolts.

2) Attach the base plate to the linear rail system.

**Fig. 7:**
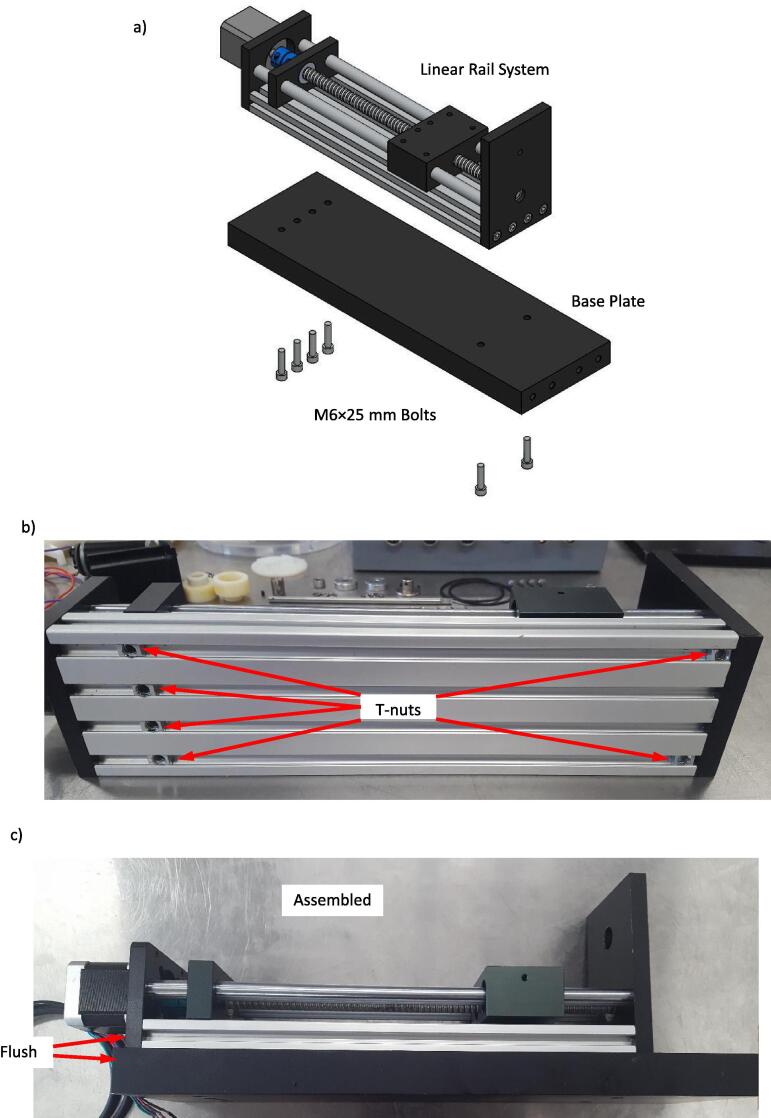
a) Exploded view showing the base plate assembly, b) underside of linear rail system with t-nuts and c) assembled base plate photo.

Orientate the base plate with the 4 countersunk holes towards the stepper motor. The rear of the base plate should be approximately flush with the stepper motor mount of the linear rail system. Align the aluminium extrusion t-nuts (Fig. 7b) with the countersunk holes in the base plate. Use the six M6 × 25 mm bolts to secure the Base Plate to the linear rail system (Fig. 7a). Once assembled the base should lay flat from the countersunk holes (Fig. 7c).

3) Attach the slotted plate to the base plate.

**Fig. 8:**
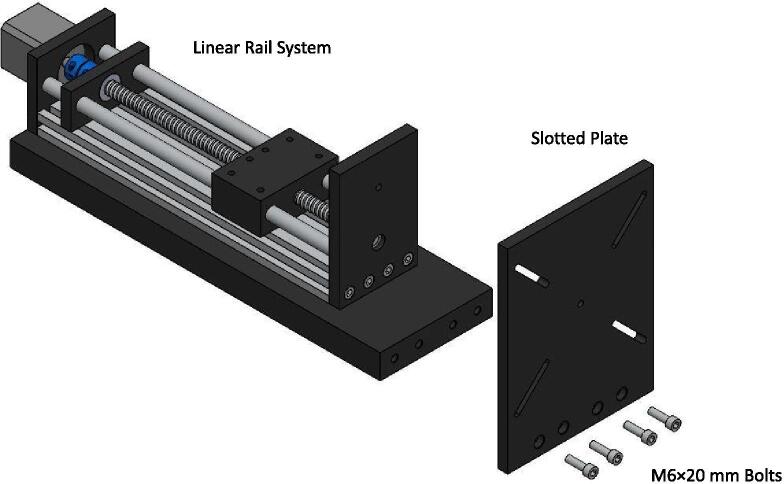
Exploded view showing assembly of the slotted plate.

Orientate the slotted plate with the four countersunk holes facing away from the base plate. Fix the slotted plate in place using the four M6 × 20 mm bolts (Fig. 8). The front face of the base plate should be machined flat, allowing the slotted plate to be fixed at 90° to the base.

4) Attach the motor to the motor mount.

**Fig. 9:**
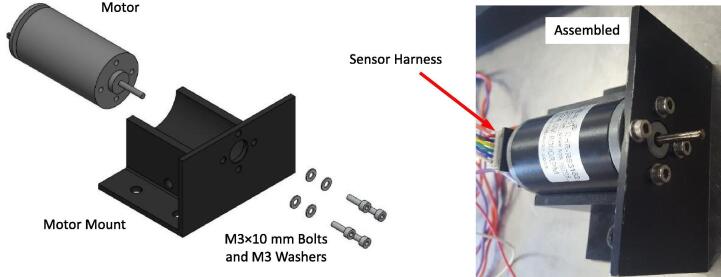
Exploded view showing motor mount assembly and photo of assembled components.

Sit the DC motor in the cradle of the motor mount and slide forward until the front face of the motor meets the front plate of the motor mount. Rotate the motor to align the 4 fixing holes on the motor mount to those on the motor. Preferably, the hall effect sensor harness socket is facing upward for easy access. Fix in place using the four M3 × 10 mm bolts with M3 washers (Fig. 9).

5) Fix the motor mount to the linear rail system.

**Fig. 10:**
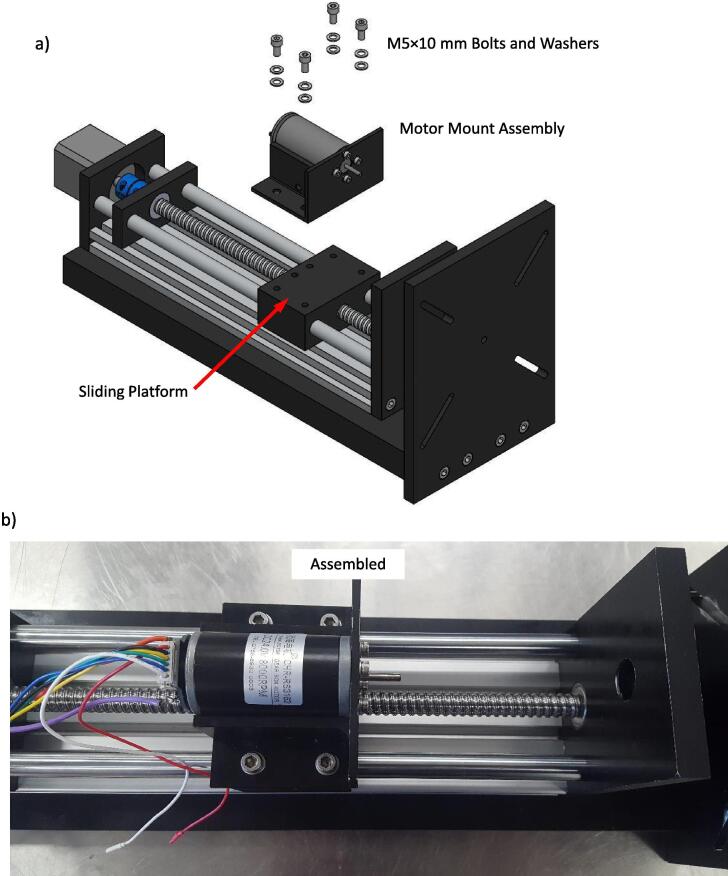
a) Exploded view showing assembly of the motor mount to the linear rail system and b) photo of assembled motor mount and linear rail system.

Sit the motor mount on the sliding platform of the linear rail system with the motor shaft facing the slotted plate. The holes in the motor mount base align with those on the sliding platform. Fix in place using the four M5 × 10 mm bolts and M5 washers (Fig. 10). In this case use two washers to ensure that each bolt is secured firmly without interference. This could simply be avoided by using shorter bolts, however, were unavailable at the time of purchase.

6) Insert bearings.

**Fig. 11:**
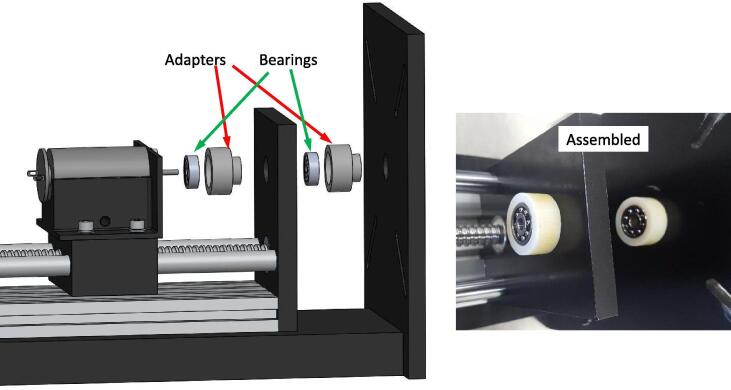
Exploded view of bearing adapter assemblies and photo is assembled components.

Press the self-aligning bearings into the 3D printed bearing adapters. Self-aligning bearings were utilised to accommodate any misalignment, to which the preliminary test results were very sensitive. The 3D printed adapters were used to allow for easy removal of the bearings for regular maintenance such as cleaning and lubrication due to their open-type nature. The adapters are pressed into the corresponding positions in the bearing plate and slotted plate in line with the motor shaft (Fig. 11).

7) Attach motor coupling

Due to the lack of component availability at the time of construction, a small 3D printed sleeve (3 mm ID, 5 mm OD with an M3 grub screw hole) was required to fit the 5 mm cup joint to the 3 mm motor shaft. The sleeve is pressed into the cup joint and the grub screw lightly tightened to sit in the sleeve hole. This allows the coupling and sleeve to be pressed onto the motor shaft without the sleeve slipping. The grub screw is then fully tightened to fix the coupling.

8) Assemble the shaft components

**Fig. 12:**
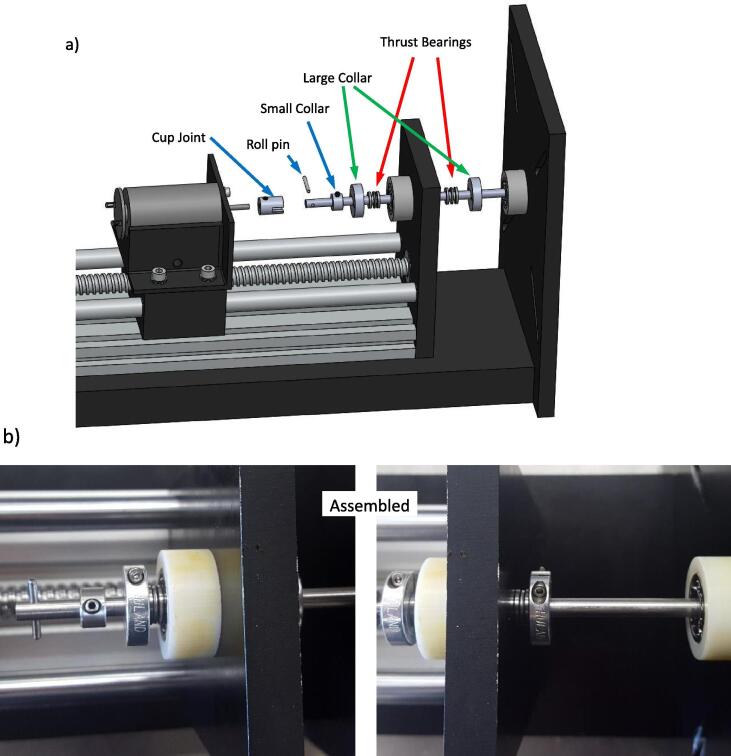
a) Exploded view showing the shaft component assembly and b) photos showing the shaft components assembled on either side of the bearing plate.

Slide the shaft through the slotted plate and front bearing assembly with the roll pin hole end first. Before reaching the bearing plate, slide on one large shaft collar and one axial thrust bearing. The thrust bearing contains two washers – the housing and shaft washer. The shaft washer fits tightly against the shaft, whereas the housing washer has a larger bore, allowing the shaft to rotate. The housing washer should be the last component (against the bearing plate). This washer will remain stationary against the bearing plate while the shaft washer is free to rotate with the large collar and shaft. Push the shaft through the bearing assembly of the bearing plate. Feed onto the shaft the remaining thrust bearing and large collar. The washer order for this thrust bearing is not important since both will be rotating with the shaft, collar and self-aligning bearing. Next slide on the small shaft collar, this is used to measure the axial positioning of the shaft (described in the operating instructions). Tap the 2 mm roll pin through the hole in the shaft (Fig. 12a). Tighten the large shaft collars to sandwich the axial bearings against the bearing plate (Fig. 12b).

9) Attach pump base

**Fig. 13:**
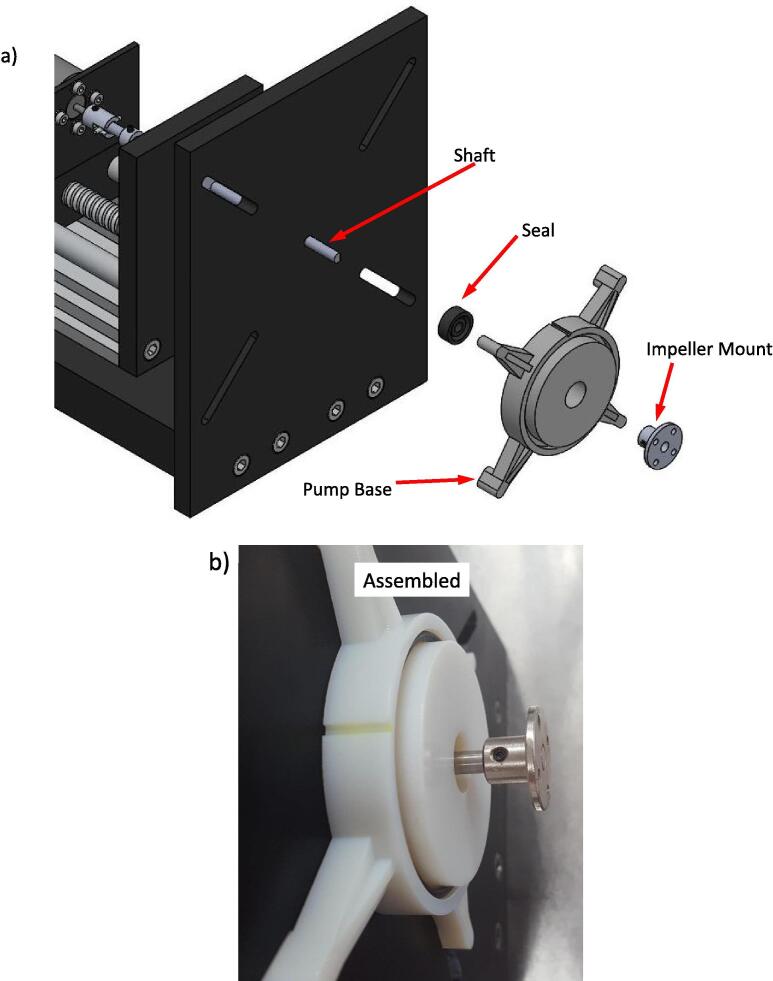
a) Exploded view showing the pump base assembly and b) photo of the assembled pump base and impeller mount.

Press the 5 mm shaft seal into the back of the 3D printed pump base with the wet side facing toward the pump assembly. Slide the pump base over the shaft via the seal and push into the slotted plate (Fig. 13a). In this case, align the pump base so that the notch is facing upward. This is done as the feet of the pump base are offset 0.3 mm to account for the misalignment from tolerance build up during assembly. This would be adjusted based on the measured misalignment. The compensation allows for the shaft to remain centred in the pump. Slide the 5 mm rigid flange coupling onto the D shape shaft end with the flange facing forward (Fig. 13b). This component will be used to mount the 3D printed impellers. Fix the impeller mount to the shaft using the M3 × 3 mm long grub screw on the shaft flat side and the M3 × 2 mm grub screw on the other. This allows the grub screws to secure the impeller mount against the shaft without the grub screws protruding, avoiding interference with the 3D printed pump base.

The assembly of the pump components are outlined in the operation instructions.

### 6.2 Control box

1) Cut holes in the enclosure

Mark the positions of the three GX-16 sockets, two Bayonet Neill-Concelman (BNC) connectors and DC power socket on the front (long side) of the electronics box. Ensure that the nut used to secure the sockets from the inside are not interfered with by internal structures. Drill holes the appropriate size for each socket at the marked locations. If necessary lightly file each hole to ensure a tight fit and remove any burrs. Position the touch screen on the lid located towards the left side, marking the location of the screen corners. Cut out a rectangle for the screen to fit tightly when pushed through from underneath. This can be achieved using a drill, small jigsaw and file. Position the heatsink, prototype board and stepper motor driver in the desired configuration (allow sufficient room between components) within the box and mark the positions for the standoffs. Drill these holes in the box floor for the M3 screws.

2) Fix sockets and screen

**Fig. 14:**
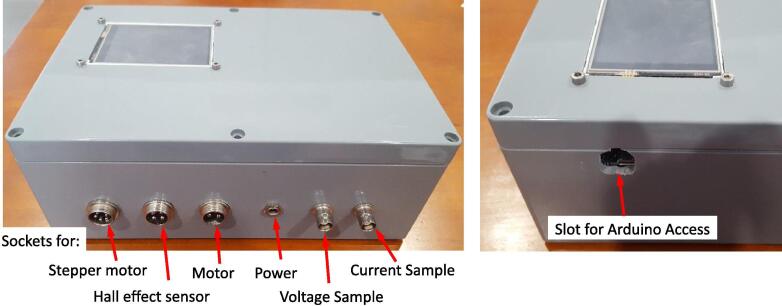
Control box socket configuration (left) and slot for Arduino access (right).

Fix each of the sockets in the appropriate front holes, held in place with the securing nuts. Attach the screen to the lid using the four M3 × 16 mm bolts, four 4 mm nylon washers (used as standoffs) and four M3 nuts. With the Arduino and shield attached to the screen, mark on the left side of the electronics box, the position of the programming port. Cut this small section from the box to allow access to the Arduino when the box is assembled (Fig. 14).

3) Connect electronics

**Fig. 15:**
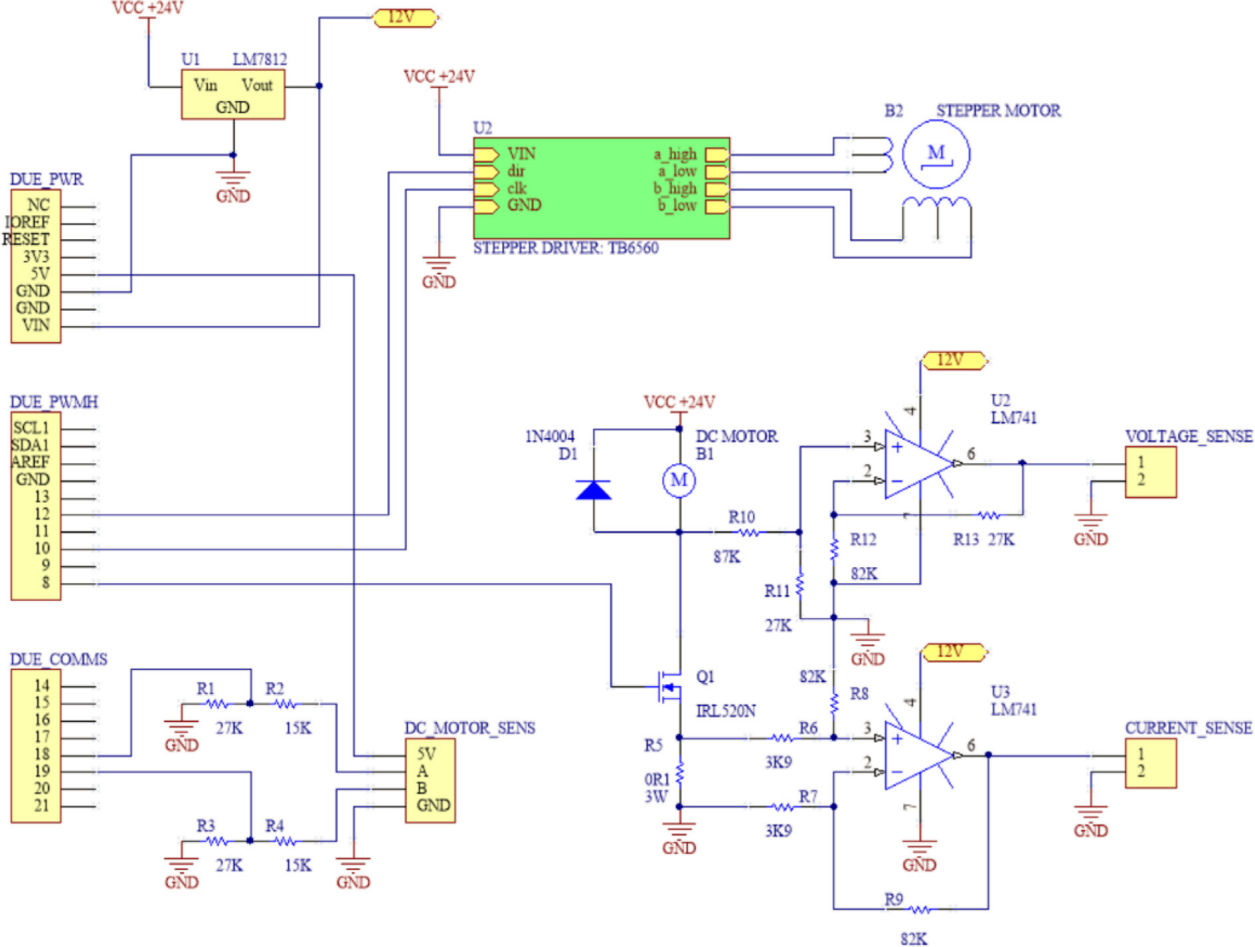
Control box electrical wiring diagram.

Connect and solder the electronic components following the wiring diagram shown in Fig. 15 (a high resolution version is available in the supplied files). Use the power cable wire (AWG 18) for connections involving the 24 V power supply and connections to the motor due to the higher expected current and the hook-up wire (AWG 26) elsewhere. Use heat shrink where possible/appropriate to avoid shorts.

Power to the motor is supplied directly from a 24 V laboratory power supply located externally to the control box. The motor speed is regulated by a PWM signal supplied by the Arduino via the N-channel MOSFET (Q1 in Fig. 15). The power supply is protected from parasitic back-EMF by D1. Signals from the motor’s hall effect sensors are connected to hardware interrupt pins (18, 19) on the Arduino via voltage dividers (R1-4) which step the signal voltage down from 5 V to 3.3 V.

The TB6560 stepper motor driver converts the logic level voltages supplied by the Arduino on the digital I/O pins (10, 12) to high current signals to drive the motor coils.

Two operation amplifiers (op-amps) were configured as differential amplifiers (U2, U3) to convert the motor voltage and voltage across the current sense resistor to appropriate levels for the DAQ interface. The rail voltage of the op-amps was set at 12 V by the use of a linear voltage regulator (U1). This regulator also supplied the voltage to the Arduino via Vin.

4) Secure components – standoffs

Feed ten Button M3 × 8 mm bolts from underneath the electrical box floor and attach the ten M3 × 25 mm standoffs (4 for the stepper motor driver, 4 for the prototype board and 2 for the voltage regulator heatsink). Position the stepper motor driver, prototype board and voltage regulator heatsink on the appropriate standoffs and secure in place using the remaining ten M3 × 8 mm bolts. Connect the 12 V voltage regulator to the regulator heat sink using the M3 × 8 mm screw connected to the standoff and a small amount of thermal paste to ensure adequate heat transfer. Connect the MOSFET to the MOSFET heat sink using the M3 × 6 mm bolt. Over time the MOSFET would gradually heat up and required a small heatsink to ensure the component does not overheat.

5) External wiring

**Fig. 16:**
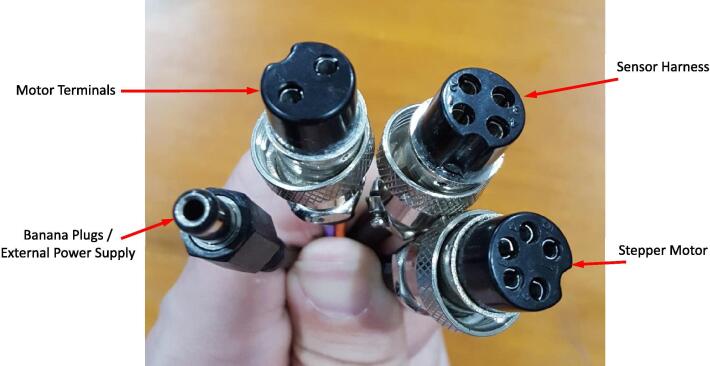
External wiring plug connections.

Using a 1 m length of the power cable wire, connect the banana plugs to one end and the DC power plug to the other. Using the remainder of the power cable wire, solder each core to the power terminals of the motor. The order of which will influence the direction of motor rotation. The other end of the power cable is soldered to the 2 pin GX-16 plug. Cut the 4-core cable into 2 × 1 m lengths. For this setup, one length of cable is soldered to the 4 pin GX-16 plug at one end and to the hall effect sensor wire harness at the other. Although there are six wires on the harness, only encoder A, encoder B, sensor power and sensor ground are connected. The remaining 2 wires represent the motor power. These are already directly soldered to the terminals which are more suited to handle higher current. The unused wires from the harness are terminated using heat shrink to ensure they do not contact other equipment. The final section of 4 core cable is soldered to the 4 wires from the stepper motor. The other end is soldered to the 5 pin GX-16 plug. One pin will be unused. This was purposefully done to ensure that the plugs could not be accidentally inserted into the wrong sockets (Fig. 16).

6) Programming

Upload the provided Arduino sketch to the Arduino Due using the Arduino IDE. The code may be manipulated to suit the desired application. The following will briefly cover the main aspects of the code.

An introduction screen was coded to appear whenever the control box is connected to power. This simply requires a touch to change to the main operating screen. This extra touch means the motor cannot be (immediately) turned on accidentally.

In order to accurately calculate the pump speed, the timer must be accurate. The internal timer, Timer 5 was accessed and used as the ‘global counter’ and set to 2.625 MHz by setting the appropriate prescalar (TIMER_CLOCK3) of 32. The global counter increased by 1 at the given frequency. Each time a signal was received from the hall effect sensor, the global counter was saved and compared to the previous value. From the difference between the values (counter difference), the known frequency of the counter and the number of hall effect pulses per revolution (11), the motor speed could be calculated.

To reduce the impact of incorrect readings, two time-based comparator algorithms are utilised. The first compares the counter difference to the previous counter difference. If the value is outside the threshold, caused by either a missed pulse or an extra false pulse, it is discarded and the previous value remains. The second comparator backs up the first thus allowing the calculated speeds from both encoders to be compared. If the difference between the speeds exceeds a threshold, the speed is discarded and the previous remains, or else the two speeds are averaged. The second comparator block filters any false readings that may have occurred within the first compare threshold. Even though the motor is running at a constant speed, the speed readings may fluctuate due to any unequal positioning of the magnet segments during motor manufacturing. A running average is used to calculate the average speed over 10 rotations (110 pulses).

The calculated speed is used as feedback for the PID controller. The gains were tuned as outlined in section 7.4. The controller would adjust the speed by varying the duty cycle of the PWM signal. To allow for fine speed adjustments, the analogue resolution was set to 12 bit.

Apart from adjusting the motor speed, the touch screen is also used to change the position of the motor mount by controlling the stepper motor. Using the 1204 ball screw, the number of steps were set for each touch to result in 10 μm of movement. By selecting the bottom left of the screen, the step size can be changed to 1 mm movement steps for coarse adjustment.

### 6.3 Test loop

1) Reservoir

**Fig. 17:**
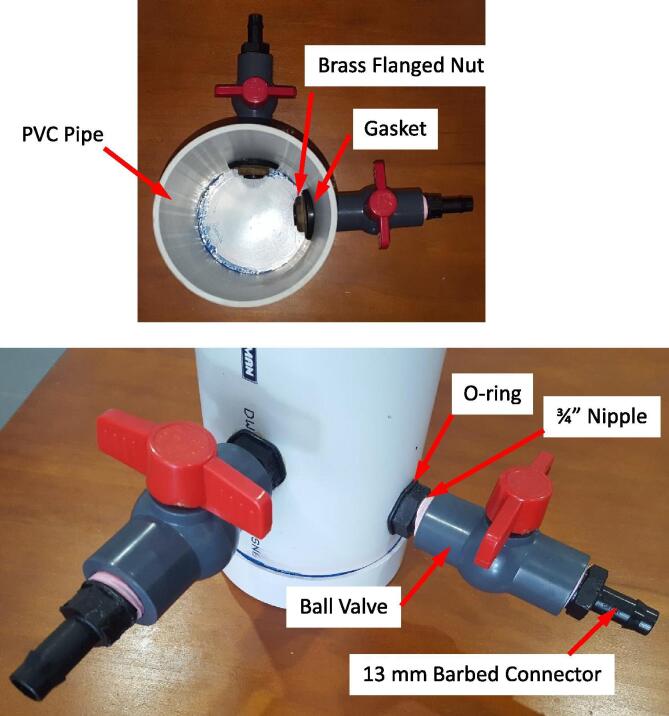
Photos of assembled reservoir showing the internal (top) and external (bottom) components.

Cut the PVC pipe to a length of approximately 250 mm. Prime and glue the PVC cap on one end, forming the bottom of the reservoir. Drill two 20 mm diameter holes, 55 mm and 95 mm from the base, 90° apart (with the higher hole in a clockwise position relative to the lower). Slide the 18 mm o-ring over the ¾” nipple and push through the drilled hole. The o-ring acts as a gasket on the outside of the reservoir. On the inside place the ¾” tank gasket (from the tank outlet) over the nipple and secure in place using the brass flange nut (top of Fig. 17). This should be tightened sufficiently to force the gasket against the inside of the pipe and the o-ring is compressed between the outside of the pipe and hex section of the nipple to ensure an adequate seal. The 20 mm ball valve is screwed onto the exposed nipple. To finalise the reservoir assembly, the 20 – 13 mm threaded to barbed connectors are wound into the free end of the ball valve (bottom of Fig. 17). Thread tape is encouraged on all threaded surfaces to assist with sealing.

2) Loop

**Fig. 18:**
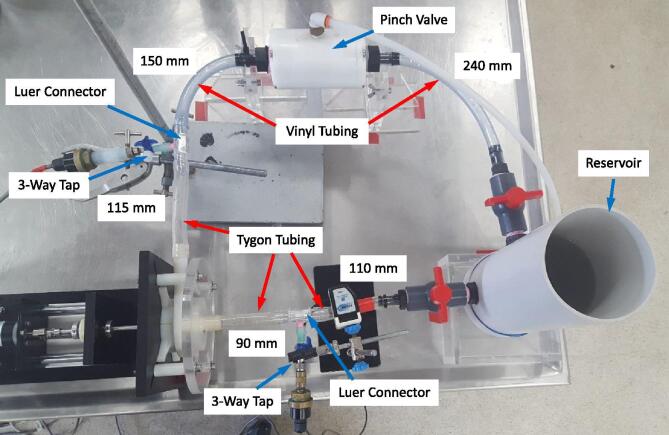
Test loop circuit showing tubing lengths.

Screw the 25–13 mm barbed connectors to each end of the pinch valve. Connect the lengths of tubing and Luer lock connectors to the reservoir and pinch valve as presented in Fig. 18. The lengths of tubing were selected so the inlet and outlet pressure sensors were positioned 125 mm from the impeller inlet and diffuser respectively. The distance equates to 10 tubing diameters to ensure that a steady velocity profile is reached, resulting in accurate pressure readings. The same principle was applied to the distance between the flow sensor and reservoir outlet hole. A 3-way tap is attached to each Luer connector and represent the pressure sampling ports. The ½” Tygon tubing (Tygon, Saint-Gobain, La Defense, France) is used due to compatibility with the flow sensor and the soft material avoids damage to the 3D printed parts. The stiffer vinyl tubing is used where the loop bends through 90° to ensure that the tubing does not kink whilst keeping the loop shorter, reducing the resistance.

3) Stands

**Fig. 19:**
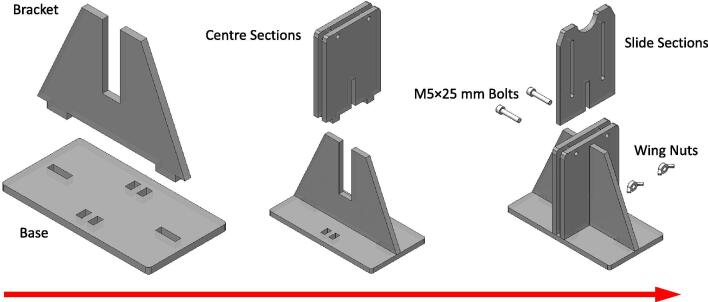
Exploded views showing the pinch valve stand assembly steps.

The reservoir was supported by an acrylic stand that was recycled from a previous test loop. The stand allows the lower reservoir opening to be at approximately the same height as the shaft. Lab stands were used to simply support the pressure transducers. Custom pinch valve stands were developed to support the pinch valve and allow height adjustment to keep the pump outflow tubing level for different volute sizes. These have been slightly adapted from the original designs of Hansi Lang. The CAD files are supplied to laser cut the stand sections. To assemble the stand, glue the bracket section along the length of the base, locking in the tabs. Slide the centre sections over the bracket and glue the tabs to the base. Finally place the slide section between the centre sections and over the bracket. Fix the slide section to the desired height by tightening the M5 × 25 mm bolt and M5 wing nuts fed through the holes and slots from the slide section. The 13 mm barbed ends attached to the pinch valve rest on the cradles of the slide section. See Fig. 19 for visualisation of the assembly steps.

4) Fluid

A water-glycerol solution is used as the working fluid to approximate the properties of blood in terms of density (1104 kg/m^3^) and viscosity (3.55 mPas). This equated to a ratio of approximately 43% glycerol by weight. Approximately 1.5 L of fluid is used in the setup.

### 6.4 Loop Control

1) Pneumatics

**Fig. 20:**
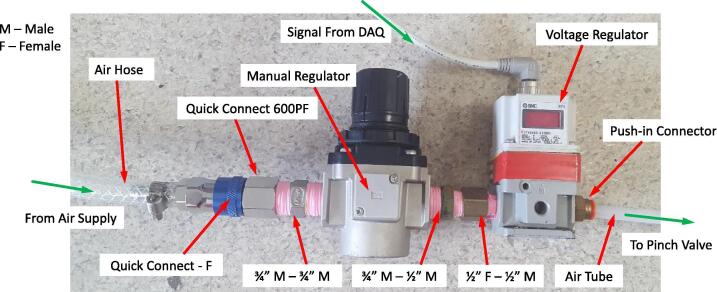
Pneumatic control assembly.

To control the flow rate of fluid around the circuit, compressed air is regulated to actuate the pneumatic pinch valve. For all threaded pneumatic connections thread tape was used to assist with sealing. Attach one male and one female air hose quick connect to each end of the PVC air hose, secured in place using the hose clamps. In our case, the male end was connected to a compressed air outlet in the lab, a small air compressor could be used as an alternative. Depending on the air supply, an alternative fitting may be necessary. Attach the 600PF quick connect to the inlet of the manual regulator via the ¾” male – male connector (Fig. 20). These components were readily available in the lab, however one of the quick connects from the six-piece set could be used if an appropriate connector to the manual regulator could be found, reducing the cost. Connect the manual regulator to the voltage regulator using the ¾” male – ½” male connector on the outlet of the manual regulator and the ½” female – male connector to the inlet of the voltage air regulator. This could be achieved with the single ¾” male – ½” male connector, however, this gave very little room between the regulators to fit the appropriate spanner. On the outflow of the voltage regulator screw in one of the push-in air tube connectors. Attach the other air tube connector to the pinch valve. Finally connect the soft nylon air tube to the push-in connectors. To control the voltage regulator a signal is supplied from the dSPACE (channel DA08).

The equipment specified was readily available in the lab. A lower-cost alternative to control the flow rate could use a stepper motor attached to a screw clamp (such as a Hoffman clamp). The stepper motor would be controlled using another Arduino and programmed PID controller receiving feedback from the flow meter. The target could be programmed on a timer in the same manner as that used by the SimuLink code.

### 6.5 Data acquisition

1) Pressures

Pressure transducers (PX181B – 015C5VX346, Omega Engineering, Norwalk, CT, USA) were connected to each 3-way tap located at the inflow and outflow of the pump. The 3-way taps allow air to be bled from the system easily as opposed to directly connecting the pressure transducer to the Luer connector. The inlet pressure and outlet pressure sensors are connected to the dSPACE through channels AD01 and AD02 respectively.

2) Flow

**Fig. 21:**
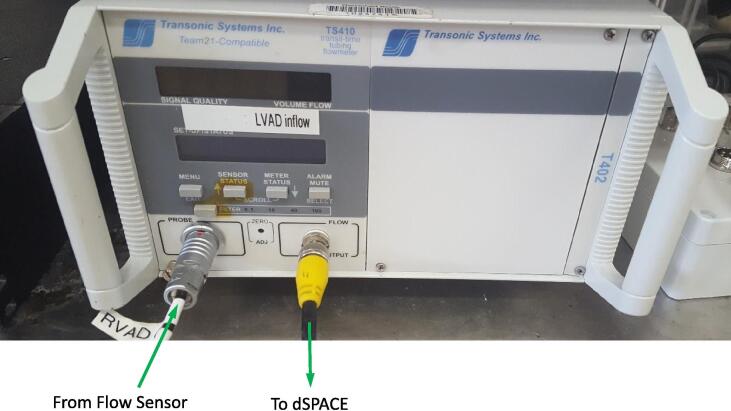
Transonic (TS410) flow meter.

To monitor the flow rate a 10PXL Transonic flow sensor (10PXL, Transonic Systems, Ithaca, NY, USA) is clipped on the Tygon tubing between the reservoir and inlet pressure transducer. The flow sensor is plugged into a TS410 Transonic flow meter (TS410, Transonic Systems, Ithaca, NY, USA), which in turn is connected to AD05 on the dSPACE via BNC cable (Fig. 21).

3) Power

To record the power consumption used by the motor, the voltage and current are connected from the control box directly to the dSPACE using BNC cables. This can be done since the op amps can only output the maximum rail voltage of 12 V, avoiding damage to the dSPACE which is protected to an overvoltage of 15 V. The voltage and current are connected to channels AD03 and AD04 respectively on the dSPACE.

4) SimuLink and Control Desk

**Fig. 22:**
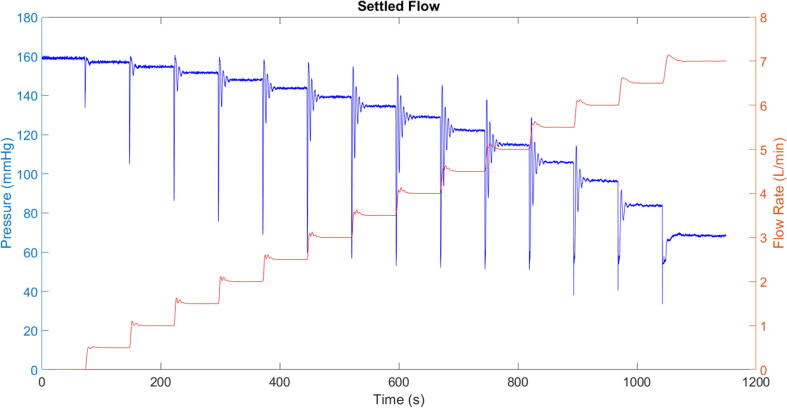
Experimental run showing sufficient settling of pressure and flow rate at each flow increment.

The provided Simulink file was developed to automate the experiment by increasing the flow rate by 0.5 L/min every 75 s. This timeframe was selected as it allows the flow rate and pressure sufficient time to settle before moving to the next target flow (Fig. 22). Control Desk (dSPACE, Paderborn, Germany) is used to display and record the data obtained from the dSPACE.

## 7 Operation instructions

**Fig. 23:**
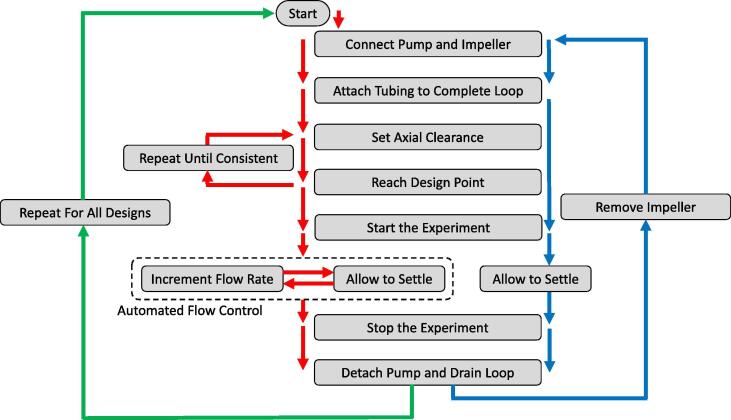
Flow chart of operation process.

The following operation instructions continue on from step 9 (attach pump base) of the build instructions for the test rig where the pump base and impeller mount have been fixed in place. An overview of the key steps of the operation procedure is presented in the following flow chart (Fig. 23).

1) Connect all external cords to the control box. Connect the banana plugs to the power supply and set to 24 V. Using the touch screen adjust the “Axial pos” to move the motor mount platform approximately halfway along the linear rail system, disengaging the cup joint and roll pin, to allow room for shaft adjustment in later steps.
2) Attach the impeller.

**Fig. 24:**
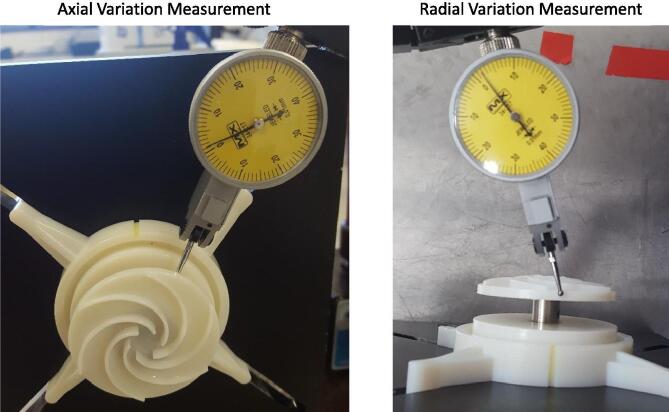
Measurement of the axial and radial variation using the dial gauge indicator.

Attach the 3D printed impeller to the impeller mount using a small amount of 5-minute epoxy. Set up a dial gauge indicator to run along the back face of the impeller near the periphery (Fig. 24). The epoxy allows some time to adjust the impeller before curing. Using the dial gauge reading, aim to reduce the axial variation as much as possible. Note this value. Once satisfied, set up the dial gauge indicator (MeasumaX 34–217, MeasumaX, Hare & Forbes Pty Ltd, Northmead, Australia) to run along the periphery face of the impeller and measure the amount of radial deviation. If this is outside of the acceptable range, the impeller is removed and requires reprinting. Take care when using the dial gauge indicator as any sudden impacts may strain the internal spring and cause the measurements to become inaccurate.

3) Once the epoxy has set, loosen the large shaft collars and set the position of the shaft so that there is a small gap between the back impeller face and pump base (<0.5 mm). Tighten the large collars against the bearing plate to secure the shaft position. This ensures no interference between the pump top and impeller during assembly.
4) Fit the printed pump components.

**Fig. 25:**
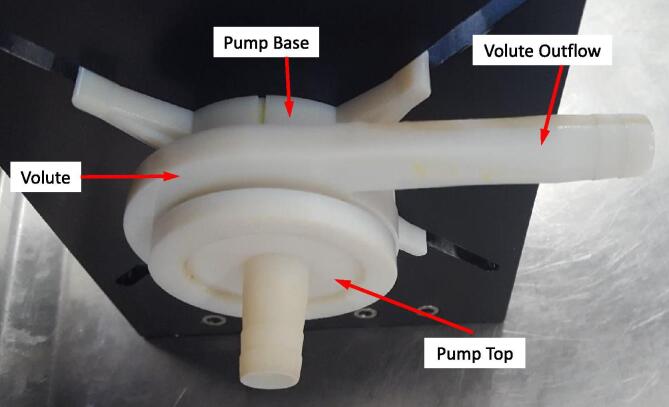
3D printed pump assembly.

Fit the 52 mm o-rings, one each in the groove of the pump base and in the groove of the pump top 3D printed parts. Mate the 3D printed volute to the pump base where the step aligns with the o-ring groove. Ensure that the volute outflow is aligned horizontally (Fig. 25). Mate the pump top o-ring groove to the volute step.

5) Attach full pump assembly.

**Fig. 26:**
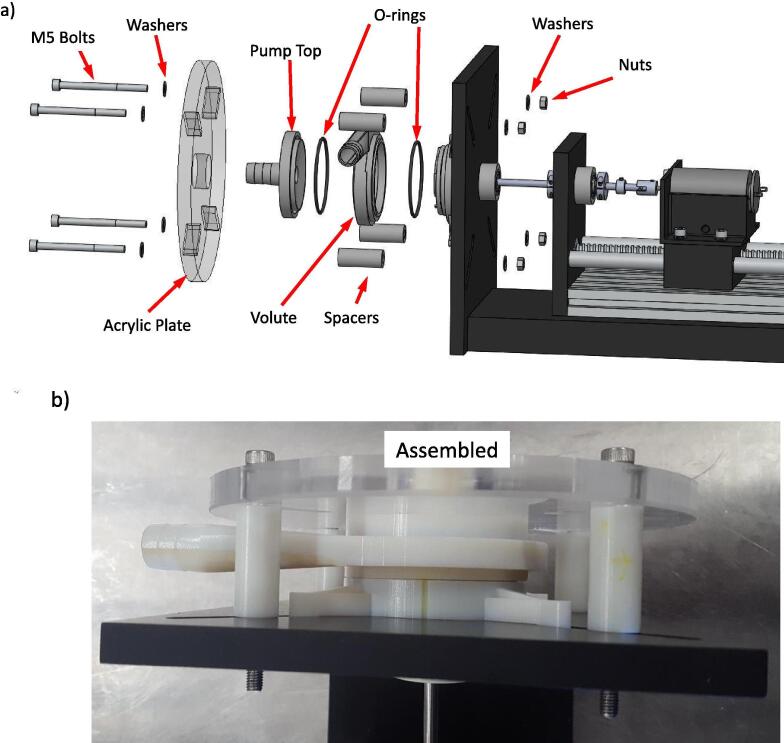
a) Exploded view showing full pump assembly and b) photo showing assembled pump components.

Place the acrylic plate over the pump inlet and against the pump top. Secure in place with the four M5 × 65 mm bolts and M5 washers using the 3D printed spacers between the acrylic plate and slotted plate (see Fig. 26a). The spacers are used to ensure that the assembly is not overtightened and apply equal pressure. It is highly recommended that a torque wrench is used to ensure the bolts are tightened consistently. The torque wrench was set to 1 Nm. The bolts should be tightened in an opposing pattern to avoid warping of the printed parts and apply equal pressure around the pump assembly (Fig. 26b).

6) Connect the Tygon tubing from the lower port of the reservoir to the inlet of the pump. Attach the Tygon tubing leading to the pinch valve to the pump outflow. Fill the reservoir with the water-glycerol solution. Slowly open the reservoir taps and allow the loop to fill. Slightly raise the reservoir and tilt the pinch valve to assist with filling. Stop and start the pump a few times to remove any remaining air from the system. Open the 3-way taps to bleed any air from the pressure transducers as any bubbles will interfere with the pressure readings. Turn the 3-way taps so that they are open between the pressure transducers and fluid.
7) Set the axial clearance.

**Fig. 27:**
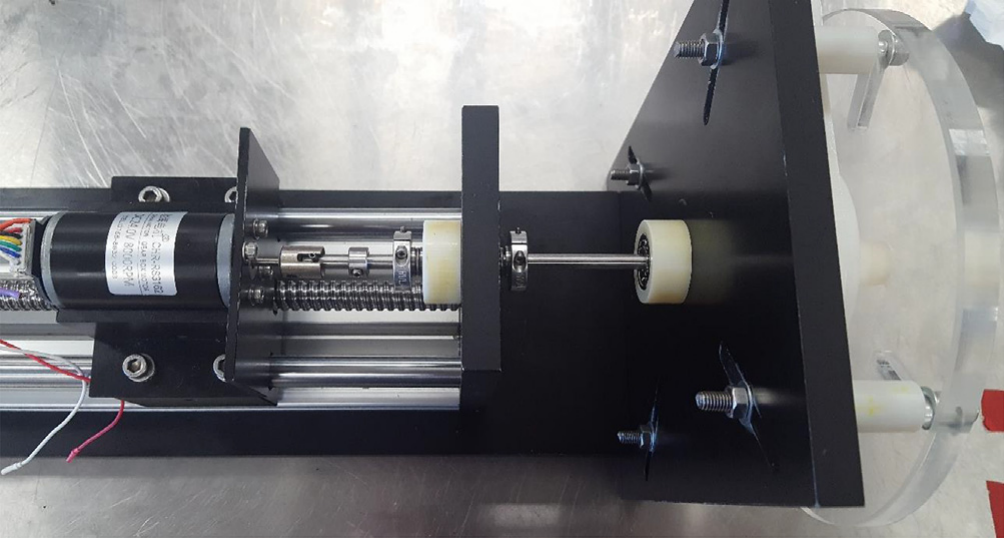
Fully assembled test rig with set shaft position.

The axial clearance between the impeller and pump top needs to be set. From here on, the large shaft collar closest to the motor will be referred to as the back collar and the large collar closest to the pump assembly, the front collar. Loosen the back collar and gently push the shaft forward while rotating the shaft by hand. When light contact is heard between the impeller and pump top, tighten the back collar and readjust the front collar to lock the shaft in place. Note the tightening and loosening of the collar screws may move the shaft slightly. If so, make the required minor adjustments. Set up the dial gauge indicator against the small collar and set the face to 0 at the needle. Loosen the front collar and gently pull the shaft away from the pump. The target distance is the desired clearance minus the variation measured from step 2. For example, for an impeller running with a variation of ± 20 μm from true and a desired clearance of 200 μm, the shaft should be positioned until the dial gauge shows 180 μm. This sets the average clearance to the desired value. Tighten the shaft collars in place, these may need small adjustments if the shaft moves during tightening of the screws. Remove the dial gauge. Using the touch screen, position the motor until the cup joint engages with the roll pin (Fig. 27).

8) Settle at a design point.

**Fig. 28:**
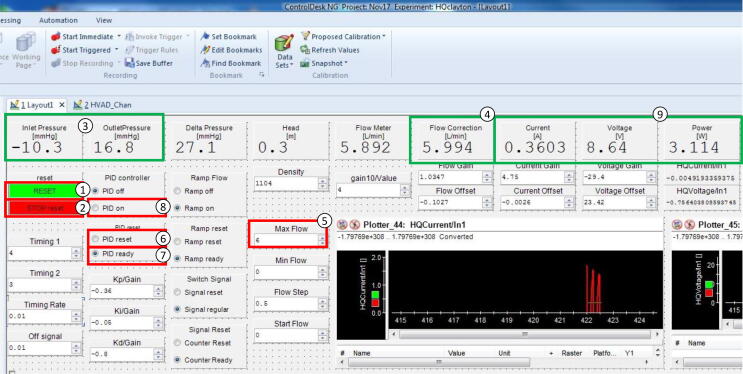
Control Desk interface with numbered steps to reach design point.

From Control Desk select “reset” (1) then “stop reset”(2) when the pressure readings reach zero (3). Repeat if the values are not hovering around zero. Turn on the motor and set the speed to the desired value using the touch screen. By default, the flow PID controller will be off and allow the maximum achievable flow rate. Refer to the “Flow Correction” (4) as this shows the calibrated flow reading. Set the “Max Flow” (5) to a value lower than the maximum observed. The “Max Flow” sets the upper limit of the allowable flow rate to ensure safe limits of the signal sent to the air voltage regulator. Click “PID reset” (6), “PID ready” (7) then “PID on” (8). This resets the integral component of the flow PID controller then turns the controller on. Allow the pressure, flow (to “Max Flow” value) and power (9) readings to settle and record these values. The button locations are shown in Fig. 28.

9) Turn off the PID controller and pump, repeat steps 7 and 8 a number of times until consistent values are recorded. This ensures that the setup is consistent, particularly the axial clearance which is very sensitive to the pump performance.
10) Once the setup is acceptable turn the PID controller off in Control Desk. Turn the pump on from the touch screen. Allow the flow to settle and note the maximum flow rate. Round down this value to the nearest 0.5 L/min and input to the “Max Flow”. Reset the flow PID controller and turn on as in step 8.
11) Slowly reduce the “Max Flow” by clicking the down arrow (0.5 L/min steps) until 0 L/min is reached. Turn off the motor. Gently remove the bearing adapter in the slotted plate and apply light redial pressure to the shaft in all directions, allowing a small amount of fluid to leak through ensuring the seal is wet. If parts of the seal run dry, the power consumption is greatly increased. Replace the bearing adapter in the slotted plate. Apply a low viscosity lubricant to the bearings. WD-40 (WD-40, San Diego, CA, USA) was used as it produced the most repeatable power consumption results. Turn on the motor and allow the readings to settle at 0 L/min flow rate.
12) Start the experiment.

**Fig. 29:**
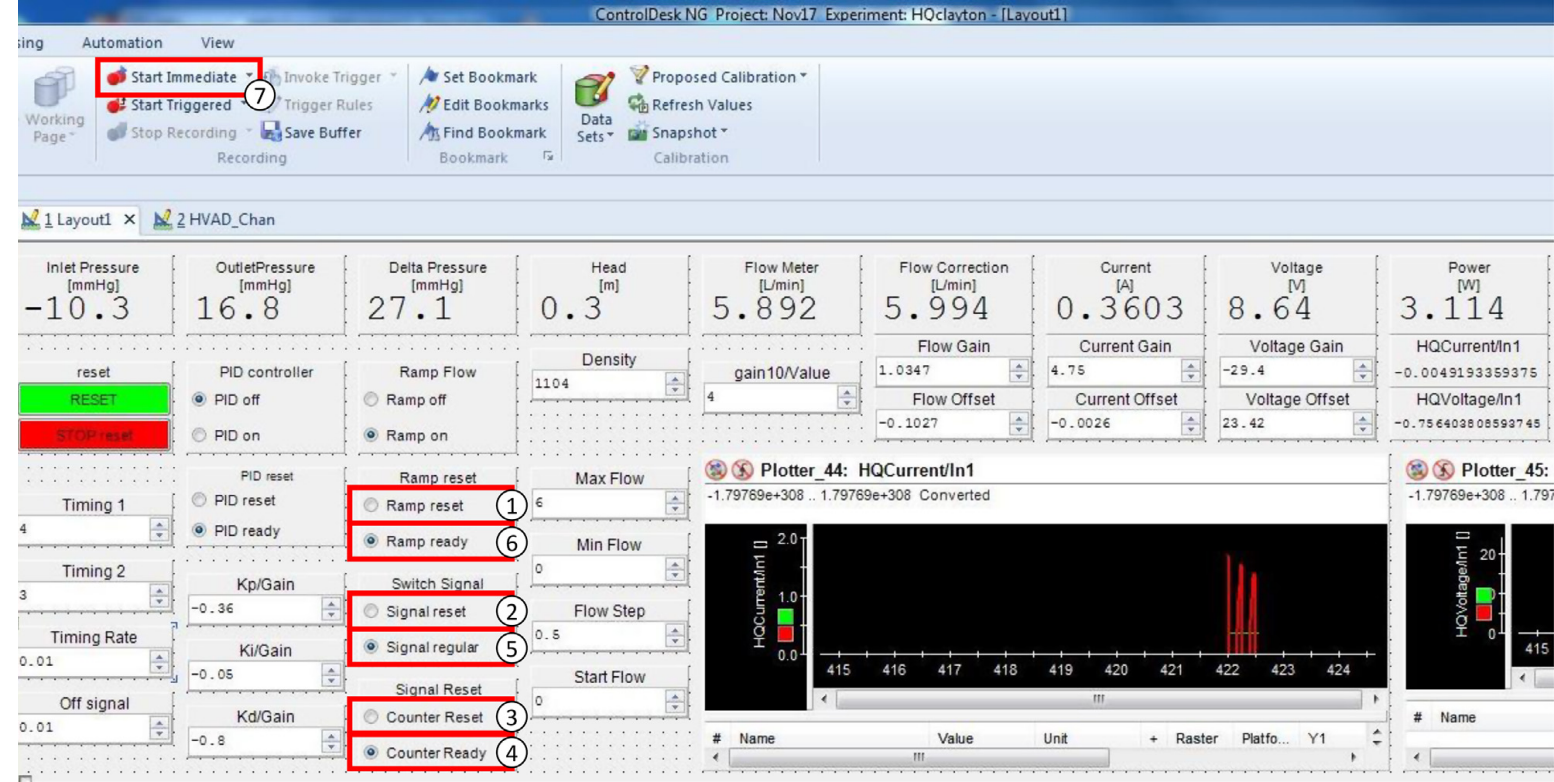
Control Desk interface with numbered steps to start the experiment and record data.

To start the test, click on “Ramp reset”(1), “Signal reset”(2) and “Counter reset”(3). Then quickly select “Counter ready”(4), “Signal Regular”(5), “Ramp ready”(6) then “Start Recording”(7) in the given order (Fig. 29). This resets all timing variables for the experiment and starts the recording.

13) Before the first step is complete (75 s) set the “Max Flow” to a value of 0.2 L/min lower than the maximum achieved flow rate from step 10.
14) Stop the experiment.

**Fig. 30:**
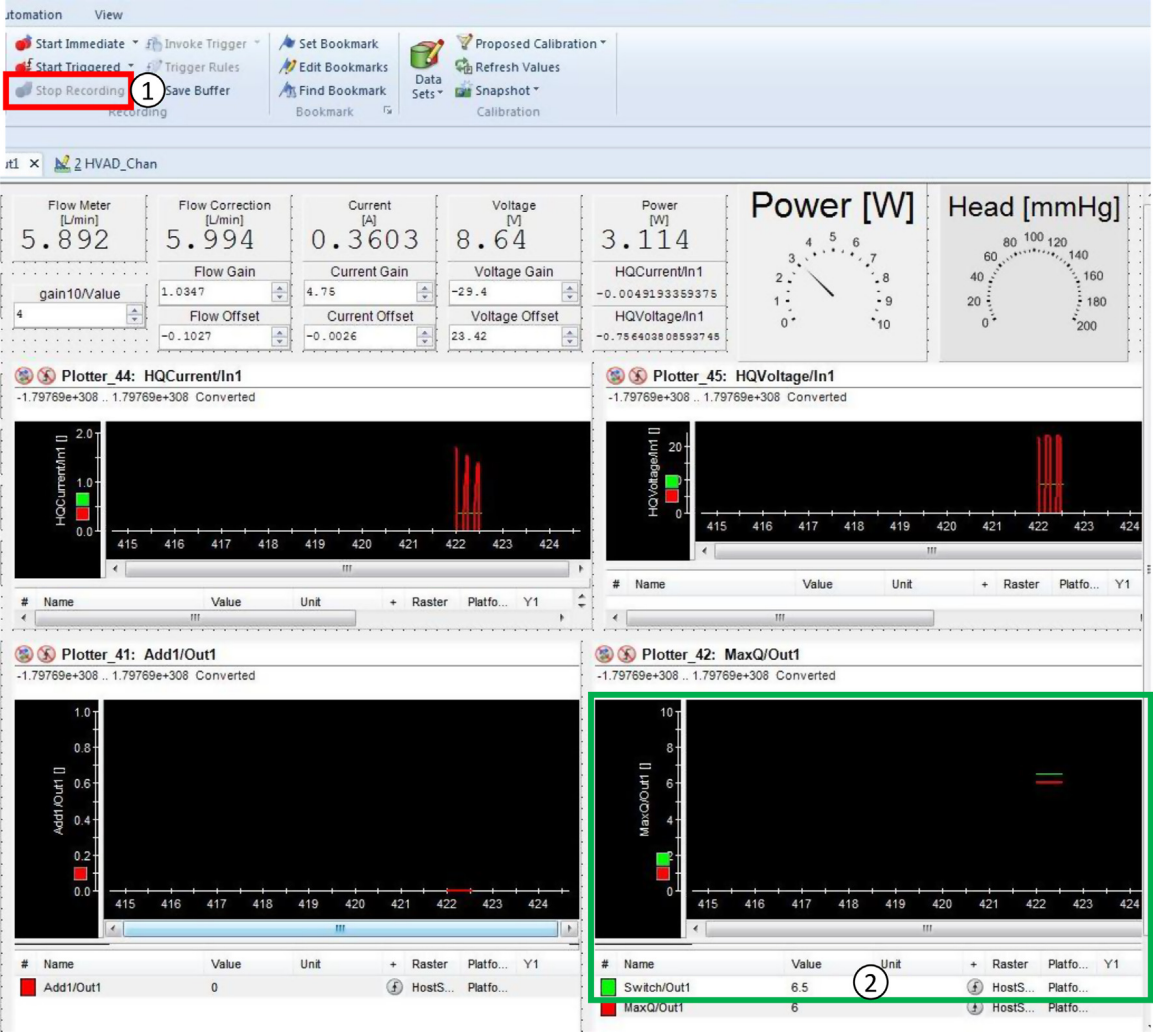
Control Desk interface with numbered steps to end the experiment.

Click stop recording (1) after the flow rate signal (2) has reached a step higher than the value set in step 13 rounded up to the next 0.5 L/min. For example, if the maximum flow rate is set to 8.3 L/min, stop the recording at or after the signal shows 9.0 L/min (Fig. 30). This ensures that data has been recorded for the final data point. Turn off the flow PID controller and the motor.

15) Close the reservoir taps. Place a small container under the pump assembly to collect the fluid draining from the loop. Remove the Tygon tubing from the outlet and inlet of the pump. Undo the nuts and bolts from step 5 and remove the pump top from the volute.
16) Loosen the back collar and slide the shaft forward to allow access to the impeller mount grub screws. Gently pry the impeller off the impeller mount and remove any residual epoxy. Loosen the grub screws and remove the impeller mount from the shaft. Slide the shaft back into position and tighten the back collar to secure in place.
17) Repeat steps 4, 5 and 6 (without the impeller or impeller mount). Wet the seal and lubricate the bearings as in step 11. Set the pump speed to that used in the experiment and turn on the motor. Click “Start immediate” (starts recording) and approximately 60 s later select “Stop Recording”. The voltage and current readings recorded indicate the power consumed by the test rig (bearings, seals, motor windings, etc.) in order to run the pump at the desired speed.
18) Turn off the motor and drain the loop as in step 15. Reattach the impeller mount to the shaft.
19) Repeat all steps for impellers to be examined.
20) Run the MatLab script to analyse the data and generate P-Q and efficiency curves.

## 8 Validation and characterization

### 8.1 Flow rate calibration

A simple bucket test was used to calibrate the flow rate readings for the working fluid using the 10PXL flow sensor and Transonic flow meter. The test rig setup outlined above was used. Two longer lengths of ½” Tygon tubing were connected to the pump inlet and outlet. The reservoir consisted of a bucket (bucket 1) with approximately 6 L of working fluid. Another bucket (bucket 2) was placed on a set of scales and zeroed. A small Hoffman clamp was used to add resistance and dictate the flow rate. The tubing from the pump outflow was quickly transitioned to bucket 2 and the stopwatch started. The flow rate shown in Control Desk was recorded. Just before bucket 1 is emptied, the outflow tube was transitioned back to bucket 1 and the stopwatch stopped. The mass showing on the scales was recorded.

**Fig. 31:**
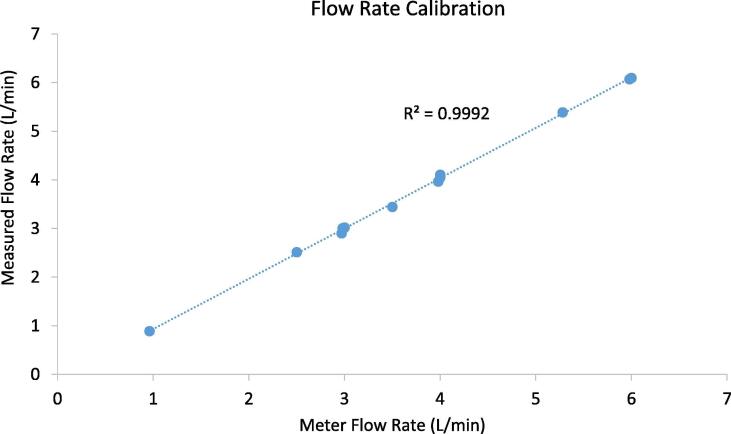
Flow rate calibration data points with line-of-best-fit.

Using the measured density (1104 kg/m^3^), time and mass, the actual flow rate can be calculated. The actual flow rate and the readings from Control Desk were recorded. The fluid from bucket 2 was transferred to bucket 1. Bucket 2 is replaced on the scales and re-zeroed to account for any residual fluid. The Hoffman clamp was adjusted to alter the flow rate and the process repeated. The measured data was plotted against the actual flow rate (Fig. 31) and the equation for the line of best fit noted. These values provide the gain and offset for the flow rate (Table 1). To validate, the new gains were input into Control Desk and obtained data for additional points, ensuring they matched the actual flow rate.

**Table 1:** Calculated flow rate gain and offset.

	Gain	Offset
Flow Calibration	1.0347	−0.1027

### 8.2 Power calibration

In a similar fashion to the flow rate, the voltage and current readings must be calibrated to give accurate power recordings. The gains calculated from the resistors in the op amp circuits could not be used as the ADC of the dSPACE had input impedance which altered the output voltage of the op amps The test loop was setup as outlined in the build instructions and operation instructions. The PID controller for the pinch valve was kept off (PID gains had not yet been determined), with the flow rate adjusted using a Hoffman clamp. Initially the pump speed and flow rate were set relatively low. The voltage and current values shown on Control Desk were recorded. The actual voltage was measured at the motor terminals using a multimeter (DigiTech Cat: QM-1320, Harman International Industries, Stamford, USA). The current passing through the motor was determined by measuring the voltage across the current sense resistor (using the DigiTech multimeter) and calculated using the formula I = V/R, where V is the voltage across the resistor and R is the value of the resistor (0.1 ± 1% Ω).

**Fig. 32:**
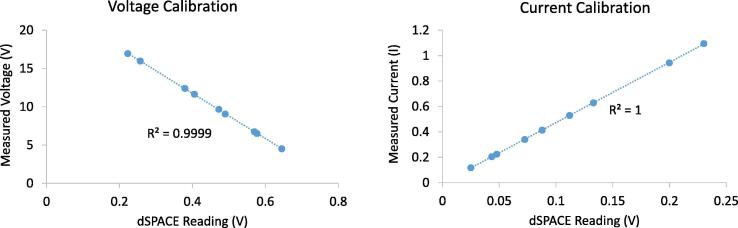
Voltage (left) and current (right) calibration data points with lines-of-best-fit.

The Hoffman clamp was slightly released to allow a higher flow rate and hence higher load on the motor. All necessary values were recorded and the process repeated for multiple flow rates. The process of adjusting the flow rate was repeated for different pump speeds. The recorded data was plotted against the actual results and applied a line of best fit (Fig. 32). From the linear regression formula, the new gain and offset values for the voltage and current were input to Control Desk (Table 2). The new gains were checked by validating for a few different points. Additional data points may be required until satisfied that the recorded values match the actual values obtained from the multimeter.

**Table 2:** Calculated gains and offsets for the voltage and current measurements.

	Gain	Offset
Voltage Calibration	−29.4	23.42
Current Calibration	4.75	−0.0026

### 8.3 Pressure sensor calibration

The pressure sensors used had been calibrated previously. To ensure the readings were still accurate, they were compared to those obtained using an NIST (certificate) calibrated pressure gauge (DPG1001B-15G, Omega Engineering, Norwalk, CT, USA). The pressure gauge was connected to the 3-way tap at the pump outlet along with one of the pressure sensors. The pump speed was increased and resistance added via the pinch valve in order to increase the pressure at the sampling point. The readings matched between the sensor and pressure gauge, confirming the sensor readings were still valid. The test was repeated with the second pressure sensor, which also produced validated measurements.

### 8.4 PID controller tuning

**Fig. 33:**
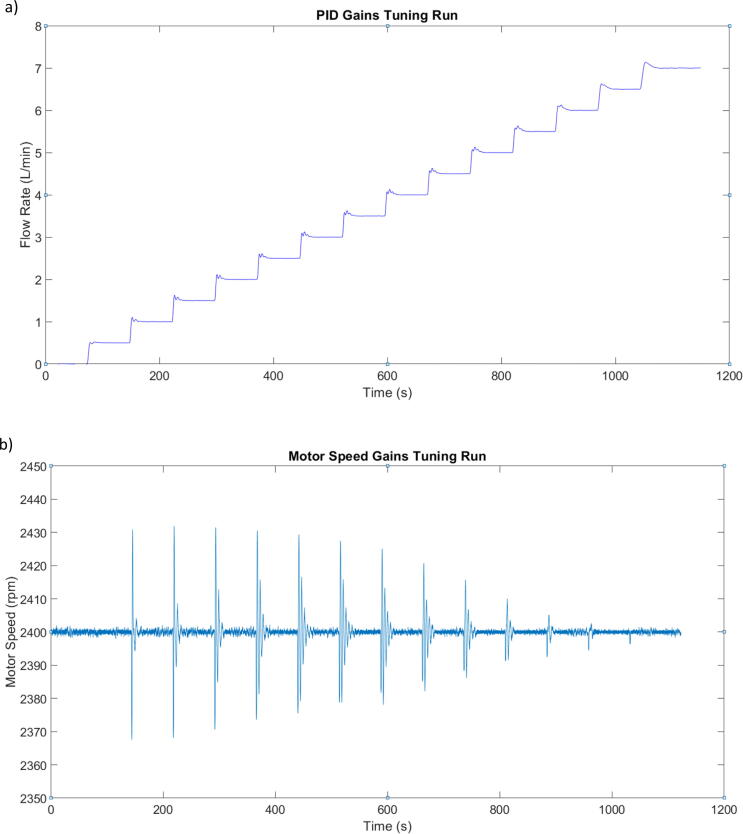
Experimental runs showing sufficient settling and stability of a) flow rate and b) motor speed PID gains.

Tuning for each of the PID controllers used for pump speed (Arduino) and flow rate (dSPACE) could not be tuned individually. Since the pump speed effects the flow rate and vice versa (through pump load) the gain values were determined experimentally. The objective was to find a good balance between the settling time of the flow rate and maintaining pump speed when the target flow rate was changed by ± 0.5 L/min. The final values are presented in Table 3. Fig. 33 shows the flow rate and motor speed traces for an experimental run demonstrating the acceptable settling characteristics. Note the timing between the graphs is mismatched due to the motor speed being recorded from a different platform (Arduino serial port).

**Table 3:** Experimental tuning values used for the flow rate and pump speed PID controllers.

	Flow Rate (SimuLink)	Pump Speed (Arduino)
K_P_	−0.36	0.09
K_I_	−0.05	0.0001
K_D_	−0.8	0.06

All calibration and tuning gains were hard-coded into the Simulink file. Therefore, upon start-up, the gains from Table 3 are the default values.

### 8.5 Setup repeatability

To determine the repeatability of the setup, an impeller and volute were designed with the following parameters (Table 4) and is available in the supplied files.

**Table 4:** Repeatability pump design parameters.

	Design Value
Impeller Diameter	50 mm
Eye Diameter	10 mm
Outlet Angle	22.5°
Inlet Angle	35°
Inlet Height	4.3 mm
Outlet Height	1.5 mm
Number of Blades	6
Volute Design	Constant Mean Velocity
Axial Clearance	0.4 mm

The impeller, volute and pump top (STL files provided) were 3D printed. Four identical copies of the impeller were printed to take into account any variation from the 3D printer. The first impeller was attached and the procedure followed as outlined in the operation instructions up to step 14. This starts the flow rate at 0 L/min and increases until the maximum flow is reached. The second test ran in reverse, decreasing the flow rate from maximum to 0 L/min. The purpose was to examine if any hysteresis exists in the circuit.

**Fig. 34:**
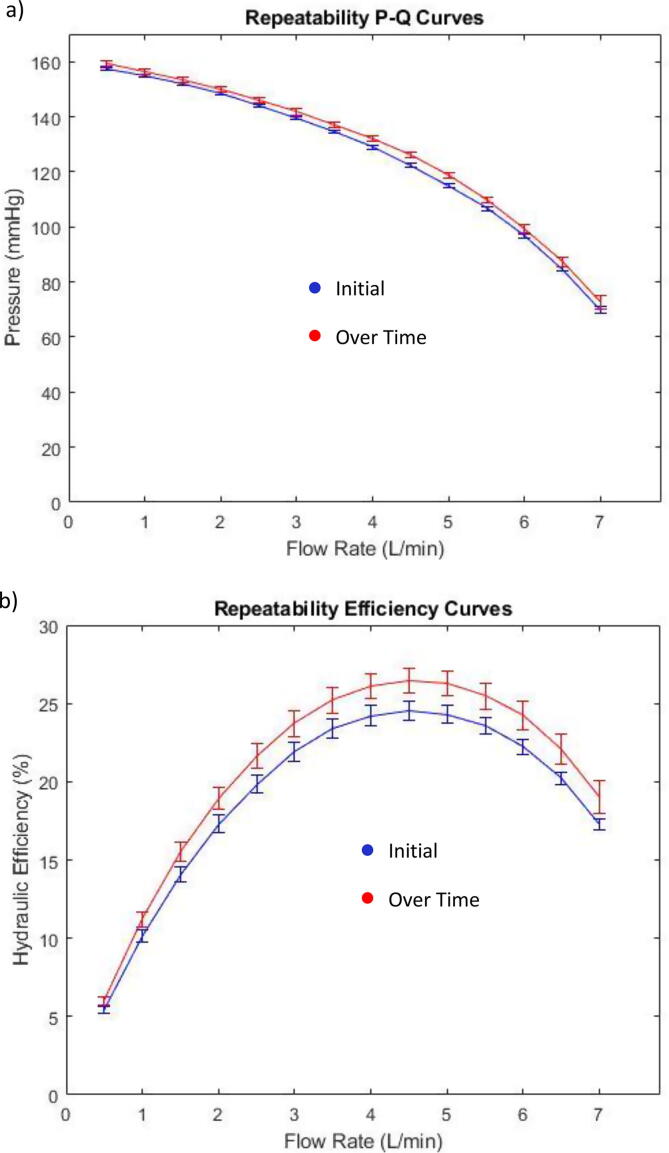
Repeatability results for a) P-Q curves and b) efficiency curves from the initial repeatability tests (blue) and over time (red) with standard deviation error bars.

The housing was then removed as in step 15 and reattached to introduce any variation in the pump assembly setup. The previous two experiments were then repeated by increasing and decreasing the flow rate. These (four previous) experiments were repeated for all four impeller prints, resulting in 16P – Q and efficiency curves. The average standard deviation for the P – Q curves was 0.7 mmHg and 0.46% for the efficiency curves (Fig. 34). These results are deemed well within acceptable bounds.

For these repeatability tests, the detected pump speed was output via the Arduino serial port. The results showed that during the settled periods (data time intervals used to plot the curves) the speed varied by a maximum of ± 2 rpm with the vast majority of readings falling within ± 1 rpm. This confirms the PID gains for the speed controller are acceptable.

### 8.6 Repeatability over time

Each day data was collected for a single run of the “repeatability” pump before gathering data for various pump designs. This allowed the performance parameters to be tracked over time (3 months, n = 38) as well as ensure that the test setup was functioning correctly.

The average standard deviation for these P – Q curves was 1.2 mmHg and 0.7% for the efficiency curves (Fig. 34). These variations are higher than those from the initial repeatability tests. Likely causes are the component wear over time and simply due to the larger number of experiments (n = 16 vs. n = 38). The mean P-Q curves over time are on average 2.5 mmHg higher than the initial tests and efficiencies 1.57% higher.

The difference in pressure places the initial (average) P – Q curve approximately two standard deviations below the (average) P – Q curve over time. The initial tests were performed using the same fluid. Before each ‘check’ repeatability test the fluid was adjusted to maintain a viscosity of 3.55 ± 0.05 mPas. The viscosity was measured using a Brookfield DV2T viscometer with a Brooks and Wells cup and plate attachment having an accuracy of 0.06 mPas. Small variations in the fluid properties would slightly influence the pressure generated by the pump, reflected in the results obtained.

The power usage in the initial tests was slightly higher (resulting in lower efficiency), likely caused by using a new seal. Whenever a new seal was required for following tests, it was allowed to run for approximately 8 hrs to properly wear in. The new seal adds more load to the shaft. Although this extra power is negated by subtracting the power usage without the impeller (step 17 of the operation instructions), the additional load causes the motor to heat up, increasing the resistance and hence the power drawn. The current setup could be improved by introducing a temperature probe on the motor to account for these additional losses.

The purpose of the repeatability ‘check’ tests was to ensure correct functioning of the system and identify any component issues. Over the 3 month testing period which the test rig was utilised, only the seal required replacement. This was necessary approximately every 25 h of testing (excluding the 8 h run time to wear in the seal which was required with every seal replacement) to maintain acceptable accuracy levels. In the event of component failure, the ‘check’ test results would reflect the presence of an issue. The modular design of the system allows for easy replacement of components. The longevity of the system is maximised through the selection of high quality components and regular maintenance.

## Declaration of Competing Interest

The authors declare that they have no known competing financial interests or personal relationships that could have appeared to influence the work reported in this paper.
